# The Antioxidant and Anti-Inflammatory Activities of the Methanolic Extract, Fractions, and Isolated Compounds from *Eriosema montanum* Baker f. (Fabaceae)

**DOI:** 10.3390/molecules29245885

**Published:** 2024-12-13

**Authors:** Gaétan Tchangou Tabakam, Emmanuel Mfotie Njoya, Chika Ifeanyi Chukwuma, Samson Sitheni Mashele, Yves Martial Mba Nguekeu, Mathieu Tene, Maurice Ducret Awouafack, Tshepiso Jan Makhafola

**Affiliations:** 1 Centre for Quality of Health and Living, Faculty of Health and Environmental Sciences, Central University of Technology, Bloemfontein 9300, South Africa; tgaetan@cut.ac.za (G.T.T.); enjoya@cut.ac.za (E.M.N.); cchukwuma@cut.ac.za (C.I.C.); smashele@cut.ac.za (S.S.M.); 2 Natural Products Chemistry Research Unit, Department of Chemistry, Faculty of Science, University of Dschang, Dschang P.O. Box 67, Cameroon; yves.nguekeu@univ-dschang.org (Y.M.M.N.); mathieu.tene@univ-dschang.org (M.T.)

**Keywords:** inflammation, antioxidant, anti-inflammatory, *Eriosema montanum*

## Abstract

**Background:** Inflammation is a natural body’s defense mechanism against harmful stimuli such as pathogens, chemicals, or irradiation. But when the inflammatory response becomes permanent, it can lead to serious health problems. In the present study, the antioxidant and anti-inflammatory potentials of the *Eriosema montanum* methanolic extract (EMME), as well as its isolated fractions (FA-FJ) and compounds (**1**–**7**), were evaluated by using in vitro and cellular models. **Methods:** The total phenolic and flavonoid contents were determined using, respectively, Folin–Ciocalteu and aluminum chloride colorimetric methods, while 2,2′-azinobis-(3-ethylbenzothiazoline-6-sulfonic acid (ABTS), 2,2′-diphenyl-1-picrylhy-drazyl (DPPH), and ferric ion reducing antioxidant power (FRAP) were used to determine the antioxidant activity. Thin Layer Chromatography (TLC) and column chromatography (CC) were used to isolate and purify the compounds and their elucidation using their NMR spectroscopic data. **Results:** *EMME* had moderate antioxidant and anti-inflammatory activities, while fraction FF showed much higher efficacy with IC_50_ values of 34.64, 30.60, 16.43, and 77.29 μg/mL against DPPH, ABTS, NO, and 15-LOX inhibitory activities, respectively. The *EMME* fraction was found to be very rich in flavonoids and phenolic compounds, with 82.11 mgQE/g and 86.77 mgGAE/g of dry extract, respectively. Its LC-MS profiling allowed us to identify genistin (**5**) as the most concentrated constituent in this plant species, which was further isolated together with six other known compounds, namely, *n*-hexadecane (**1**), heptacosanoic acid (**2**), tricosan-1-ol (**3**), lupinalbin A (**4**), d-pinitol (**6**), and stigmasterol glucoside (**7**). Given these compounds, genistin (**5**) showed moderate activity against reactive oxygen species (ROS) and NO production in LPS-stimulated RAW264.7 cells compared to EMME, which suggested a synergy of (**5**) with other compounds. To the best of our knowledge, compounds (**1**), (**2**), and (**3**) were isolated for the first time from this plant species.

## 1. Introduction

The genus *Eriosema* (Fabaceae) is made up of approximately 160 species of shrubs, shrublets, and herbs widely distributed across tropical and subtropical regions of the world. *Eriosema montanum* is one of the plants from this family, which has diverse medicinal uses according to the traditional knowledge of local populations. For example, in the Democratic Republic of Congo, *E. montanum* is used as an antimicrobial and disinfectant agent and for wound healing [1]. In Burundi, the pounded fresh leaves of *E. montanum* are used for the treatment of injuries, while the leaf decoction is used as an oxytocic agent during childbirth and for the treatment of impotence, anemia, snake bite, diarrhea, dysentery, and cholera [2]. In Rwanda, the roots of *E. montanum* are used to treat conjunctivitis, cough, and pulmonary problems [3,4]. Phytochemical analyses conducted on the CH_2_Cl_2_-MeOH (1:1) of the roots, stem bark, and leaves of *E. montanum* have led to the isolation of twenty-six secondary metabolites including six isoflavonoids [genistin, genistein, malonyl genistin, isoprunetin, 7-*O*-glucopyranosyl-isoprunetin, and isoluteolin], five phenol acids [salicylic acid, 4-hydroxy-5,5-dimethyldihydrofuran-2-one, *p*-coumaric acid, eucomic acid, and 3-(4-methoxyphenyl)propanoic acid], four dihydrochalcones [montachalcone A, montachalcone B, 2′,4′,5,6′-tetrahydroxy-4-methoxy-3,3′-diprenyldihydrochalcone, and 2′,4′,4,6′-tetrahydroxy-3,3′-diprenyldihydrochalcone], three flavones [isoluteolin, isorhamnetin, and lupinalbin A], two steroids [stigmasterol and stigmasterol glucoside], one sesquiterpene [cedrol], one sugar [d-pinitol], one lactone [4-hydroxy-5,5-dimethyldihydrofuran-2-one], one pentacyclic triterpene [betulinic acid], one flavonol [quercetin], and one alkyl caffeate derivative [dodecyl-3-(3,4-dihydroxyphenyl)propenoate] [3,4]. Additionally, *E. montanum* CH_2_Cl_2_-MeOH (1:1) leaf extract showed strong activity against *Bacillus subtilis*, with a MIC value of 3.0 μg/mL [4]. In comparison, the CH_2_Cl_2_-MeOH (1:1) root extract displayed significant antiplasmodial activity, with an IC_50_ value of 17.68 µg/mL, as well as cytotoxic potential against TPH-1 cells, with a CC_50_ value of 101.5 µg/mL, thereby yielding a selective index of 5.74 [3]. Many claimed medicinal properties of *E. montanum* have not been investigated to date, and to the best of our knowledge, the antioxidant and anti-inflammatory potential of *E. montanum* has not been studied yet. In fact, oxidative stress and inflammation are recognized to play key roles in the pathogenesis of some of the diseases where *E. montanum* is applied for treatment. The search for phytochemicals that can interfere with molecular mechanisms underlying oxidative stress and inflammation can lead to the discovery of new lead compounds able to prevent or treat these medical conditions. Literature reports and previous phytochemical analyses have shown that *E. montanum* contains flavonoids and phenolic compounds that may have antioxidant and anti-inflammatory potential [1,4,5,6,7]. In the current study, the antioxidant and anti-inflammatory properties of *E. montanum* were investigated, and its phytochemical composition was characterized using liquid chromatography–mass spectrometry (LC-MS) analysis. The quantitative analysis of specific phytochemicals presented in the extract provided important insights into the composition of *E. montanum* and the potential therapeutic benefits of these identified phytochemicals. Next, fractions and compounds were isolated from *E. montanum* methanolic extract and further tested to determine their antioxidant and anti-inflammatory properties. The three fractions (F_D_, F_E_, and F_F_) were the most significantly active compared to the unfractionated extract. Among isolated compounds, genistin (**5**), identified via LC-MS as the most concentrated compound in this plant, was the only compound that was moderately effective against ROS and NO production, thereby suggesting that it might interact synergistically with other compounds to yield the biological effect of *E. montanum*.

## 2. Material and Methods

### 2.1. Plant Material and Extraction Process

The whole plant of *Eriosema montanum* Baker f. (Fabaceae) was collected in Lefock-Fondonera (West Region of Cameroon) in October 2021, and this plant was authenticated by Mr. Nana, a botanist at the Cameroon National Herbarium in Yaoundé (Cameroon) where the specimen was deposited under the voucher number 60740/HNC. The whole plant was dried at room temperature, and the dried material was ground into a fine powder. The powder of *E. montanum* (2.9 kg) was macerated for three days in MeOH (16 L × 3 times) at room temperature. After filtration through filter paper No. 1, the filtrate obtained was concentrated using a rotary evaporator to yield the crude extract (117.3 g). The percentage yield of extraction was calculated, yielding 4.06 g of extract/100 g of dry material.

### 2.2. Assessment of the Phenolic and Flavonoid Contents

The total phenolic and flavonoid contents were determined using respectively the Folin–Ciocalteu and aluminum chloride colorimetric methods previously described [8,9,10]. At the end of these experiments, the obtained results were converted into equivalents of milligrams of gallic acid (mgGAE per g of dry extract) and quercetin (mgQE per g of dry extract) for the total phenolic and flavonoid contents, respectively [11].

#### 2.2.1. Total Phenolic Contents

For this test, the resulting mixture was made using the following reagents, respectively: 20 microlitres of extract (5 mg/mL dissolved in ethanol), 100 μL of Folin–Ciocalteu reagent (1 mL of Folin–Ciocalteu in 9 mL of distilled water), and 80 μL of 7.5% (*w*/*v*) Na_2_CO_3_ solution prepared with demineralized water. Folin–Ciocalteu in 9 mL of distilled water) and 80 μL of 7.5% (*w*/*v*) Na_2_CO_3_ solution was made with distilled water. After the whole solution was incubated in the dark at 25 °C for 30 min, absorbance measurement of the solution was performed at 765 nm, with ethanol indicated in white on the SpectraMax iD3 (Molecular Devices, San Jose, CA, USA). The standard curve (y = 0.207x + 0.113; R^2^ = 0.997) obtained with a gallic acid solution (0–100 mg/L) helped to convert the absorbance values to mg/GAE (milligrams of gallic acid equivalent) per 1g of dry matter.

#### 2.2.2. Total Flavonoid Contents

The total flavonoid content (TFC) of EMME was measured using the colorimetric method of aluminum chloride. In test tubes, the reaction solution was prepared by mixing 2 mL of extract (0.3 mg in 1 mL of methanol), 0.1 mL of hexa-hydrated aluminum chloride solution (10% aqueous solution of AlCl_3_), 0.1 mL of 1 M potassium acetate, and 2.8 mL of distilled water. The reaction solution was homogenized and incubated in rooms at 25 °C for 10 min. Then, 200 μL of each mixture was transferred to a 96-well microplate, and absorbance was measured at 415 nm on a SpectraMax iD3 multimode microplate reader (Molecular Devices, San Jose, CA, USA). Absorbance was measured in milligrams of quercetin equivalents (QE) per gram of dry matter using a calibration curve (y = 7.698x + 0.237; R^2^ = 0.999) created with quercetin (0–0.1 mg/mL).

#### 2.2.3. Antioxidant Assays

Three in vitro antioxidant assays, namely, 2,2′-azinobis-(3-ethylbenzothiazoline-6-sulfonic acid (ABTS), 2,2′-diphenyl-1-picrylhydrazyl (DPPH), and ferric ion reducing antioxidant power (FRAP), were used to determine the antioxidant activity according to methods previously reported [11,12,13,14,15]. Ascorbic acid (0 to 200 μg/mL) was used as positive control in these experiments, and Formula (1) below allowed us to evaluate the percentage of radical scavenging capacity. The inhibitory concentration (IC_50_) values were evaluated by plotting a nonlinear graph of percentage radical scavenging capacity versus logarithm of tested concentrations.

Scavenging capacity (%) = [(A_0_ − A_1_)/A_0_] × 100 (1)

where A_0_ is absorbance of negative control, and A_1_ is absorbance of extract with ABTS^•+^ radical cation.

##### ABTS^•+^ Radical Scavenging Assay

Specifically, 2.45 mM of potassium persulfate and 7 mM of ABTS solution were dissolved in methanol and combined to create the ABTS^•+^ radical cation, which was stored at 25 °C for a minimum of 16 h. At 734 nm, the absorbance of the generated ABTS^•+^ was measured using a SpectraMax iD3 multi-mode microplate reader (Molecular Devices, San Jose, CA, USA) and calibrated to 0.70 ± 0.02. After carefully diluting 40 μL of EMME (1 mg/mL) with methanol and adding 160 μL of (ABTS^•+^) radical cation, the 96-well microtiter plate was left to sit at 25 °C in the dark for five minutes. Using a SpectraMax iD3 multi-mode microplate reader, the absorbance values were measured at 734 nm against the blank (extract plus methanol without ABTS^•+^). A nonlinear curve representing the percentage of ABTS^•+^ scavenging capacity vs. a logarithm of tested concentrations was then plotted to determine the inhibitory concentration (IC_50_) values.

##### DPPH^•^ Radical Scavenging Assay

To be able to determine the DPPH^•^ scavenging capacity of EMME, we performed a serial dilution of 40 μL of the sample (1 mg/mL) with methanol on a 96-well microtiter plate, with 160 μL of solution of DPPH^•^ thereafter (25 μg/mL). The incubation at 25 °C in the dark was performed after 30 min, with the absorbance values measured at 517 nm on a SpectraMax iD3 multi-mode microplate against a blank (extract plus methanol without DPPH^•^). (Molecular Devices, San Jose, CA, USA) reader. Methanol with DPPH^•^ served as the negative control. Using Formula (1), the DPPH scavenging capability was calculated at the measured concentrations above, and as previously enumerated, the IC_50_ values were also calculated, as indicated in the previously described procedure [11].

##### Ferric Reducing Antioxidant Power (FRAP) Assay

The generally used and almost standard method for performing the FRAP test is that developed by Benzie and Strain [15]. Indeed, the antioxidant power is determined in a colorimetric reaction linked to redox in which an intense blue-colored ferroustripyridyltriazine (Fe^2+^-TPTZ) complex is obtained via reduction at a low pH of ferrictripyridyltriazine (Fe^3+^-TPTZ) [14]. The incubation of a quantity of 50 μL of EMME (1.56 to 200 μg/mL) or 200 μg/mL of gallic acid (Merck, Lethabong, South Africa) on a 96-well plate was carried out with 50 μL of 1% potassium ferricyanide (prepared in 0.2 M of sodium phosphate buffer, pH 6.6) for 30 min at 50 °C. Then, 50 μL of 10% trichloroacetic acid (TCA), 40 μL of distilled water, and 10 μL of 0.1% ferric chloride were added, respectively. The absorbance values were recorded on a blank (extract plus sodium phosphate buffer) at 700 nm on a SpectraMax iD3 multi-mode microplate reader (Molecular Devices, San Jose, CA, USA); the FRAP activity was indicated as inhibition percentage of 200 μg/mL of gallic acid.

### 2.3. 15-Lipoxygenase (15-LOX) Enzyme Inhibitory Assay

The method developed by Pinto et al. [16] was used to determine the effect of the tested samples on 15-LOX inhibitory activity, with slight modifications to the 96-well microtitre plate format. Using 20 µL of tested samples at varying concentrations (3.90 to 500 µg/mL), the soybeans 15-LOX (Merck, Darmstadt, Germany) (40 µL, 200 UI/mL) were incubated at 25 °C for 5 min. The plate was then incubated at 25 °C for 20 min in the dark; after, 40 µL of linoleic acid (final concentration, 140 μM) produced in Tris-HCl buffer (50 mM, pH 7.4) was added. A total of 100 µL of the FOX reagent—sulfuric acid (30 mM), xylenol orange (100 μM), and ferrous II) sulfate (100 μM) mixed in methanol/water (9:1)—was added to terminate the test. A SpectraMax iD3 multi-mode microplate reader (Molecular Devices, San Jose, CA, USA) was used to measure the absorbance at 560 nm following 30 min of incubation at 25 °C in the dark. Gallic acid (0.78 to 100 µg/mL) was employed as the usual inhibitor of 15-LOX, while DMSO at 5% (*v*/*v*) served as the negative control. With the exception of adding the substrate after the FOX reagent, the blanks were prepared exactly like the tested samples. Formula (2) below was used to compute the 15-LOX inhibitory activity.

15 LOX inhibitory activity (%) = 100 − [(A_0_ − A_1_) − (A_2_ − A_1_)] × 100 (2)

where A_0_ is absorbance of test samples, A_1_ is absorbance of blank, and A_2_ is absorbance of negative control (DMSO 5%).

### 2.4. Assessment of NO and ROS Levels in RAW 264.7 Cells

Gallic acid (0.78 to 100 µg/mL) was employed as the usual inhibitor of 15-LOX, while DMSO at 5% (*v*/*v*) served as the negative control. With the exception of adding the substrate after the FOX reagent, the blanks were prepared exactly like the tested samples. Formula (2) below was used to compute the 15-LOX inhibitory activity in a humidified environment containing 5% CO_2_ and 95% air. For NO and ROS production inhibition assays, the cytotoxic effect of tested samples at 100 µg/mL after 24 h of exposure was initially measured on RAW 264.7 cells (10,000 cells per well) using the standard MTT assay [17]. Then, for NO production inhibition assay, RAW 264.7 cells were pre-treated with tested samples at 100 µg/mL for 2 h followed by addition of culture medium containing 500 ng/mL of lipopolysaccharide (LPS) (Merck, Darmstadt, Germany). After 24 h of incubation under standard cell-culture conditions, NO released was measured in cell supernatants using the Griess reagent (Sigma Aldrich, Darmstadt, Germany) [18], and a SpectraMax iD3 multi-mode microplate reader (Molecular Devices, San Jose, CA, USA) was used to capture the absorbance readings at 540 nm. The ability of each tested material to reduce nitric oxide synthesis by LPS-stimulated RAW 264.7 macrophages in comparison to the negative control (cells treated with DMSO at 0.5% and LPS without extract) was used to calculate the percentage of NO production inhibition. Only the most active samples were re-tested at different concentrations (100, 50, and 25 µg/mL) for NO production inhibition.

For ROS production inhibition, on a black/clear bottom 96-well plate, RAW 264.7 cells were seeded at a density of 10,000 cells per well. The cells were then pre-treated with non-toxic quantities of EMME and genistin (**5**) at 25, 50, and 100 µg/mL for two hours and then exposed to 200 ng/mL of LPS for twenty-four hours under standard culture conditions. Thereafter, the cells were washed twice with PBS, followed by the addition of 100 µL of fresh FBS-free medium containing 10 µM of the fluorescent probe 2′,7′-dichlorodihydrofluorescein diacetate (DCFH-DA) (Sigma-Aldrich, Darmstadt, Germany). After 30 min of additional incubation under usual culture conditions, PBS was added to the plate in place of the fluorescent probe. The fluorescence of the cells was measured using a SpectraMax iD3 multi-mode microplate reader (Molecular Devices, San Jose, CA, USA) at 485 nm (excitation) and 535 nm (emission). The percentage of cells treated with DMSO 0.5% without LPS, known as the negative control, was used to express the intracellular ROS levels [11]. Using an excitation/emission filter (480/535 nm) on a 40× objective, images were taken with a camera (Flexacam C1, Leica Microsystems, Wetzlar, Germany) attached to a fluorescence microscope (Leica Microsytems GmbH, Wetzlar, Germany).

### 2.5. Liquid Chromatography–Mass Spectrometric (LC-MS) Analysis

To perform high-resolution UPLC-MS analysis, two microliters of *E. montanum* extract were injected into a Waters Cyclic Quadrupole time-of-flight (qTOF) mass spectrometer (MS) that was attached to a Waters Acquity ultra-performance liquid chromatograph (UPLC) (Waters, Milford, MA, USA). Before entering the mass spectrometer, the column eluate initially passed via a Photodiode Array (PDA) detector (Waters, Milford, MA, USA), enabling the simultaneous capture of UV and MS spectra. The mobile phase consisted of 0.1% formic acid (solvent A) and acetonitrile containing 0.1% formic acid (solvent B). The gradient started at 100% solvent A for 1 min and changed to 28% solvent B over 22 min in a linear way. It then transformed to 40% solvent B over 50 s and a wash step of 1.5 min at 100% solvent B, followed by re-equilibration to initial conditions for 4 min. The flow rate was 0.3 mL/min, and the column temperature was maintained at 55 °C. Electrospray ionization was used in negative mode with a cone voltage of 15 V, desolvation temperature of 275 °C, desolvation gas at 650 L/h, and the rest of the MS settings optimized for best resolution and sensitivity. Data were acquired by scanning from *m*/*z* 150 to 1500 *m*/*z* in resolution mode and MSE mode. In MSE mode, two channels of MS data were acquired, one at a low collision energy (4 V) and the second using a collision energy ramp (40–100 V) to obtain fragmentation data as well. Leucine enkephalin was used as lock mass (reference mass) for accurate mass determination, and the instrument was calibrated with sodium formate. Separation was achieved on a Waters HSS T3, 2.1 × 100 mm, 1.7 μm column.

MSDIAL and MSFINDER (RIKEN Center for Sustainable Resource Science: Metabolome Informatics Research Team, Kanagawa, Japan) were used to perform a preliminary identification of the chemicals [19,20]. Potential compounds were selected from databases based on the precise mass elemental compositions, and they were subsequently fragmented in silico. Each potential compound match was given a score (out of 10) based on the spectral match between the in silico and measured spectra; the highest score was considered the most likely (assuming a score of at least 4). By infusing a range of catechin standards from 0.5 to 100 mg/L catechin, compounds were measured in relation to a calibration curve (y = 8312.1x + 401.63) (see Figure S17).

### 2.6. Fractionation of the Crude Extract and Isolation of Compounds

**Figure 1:**
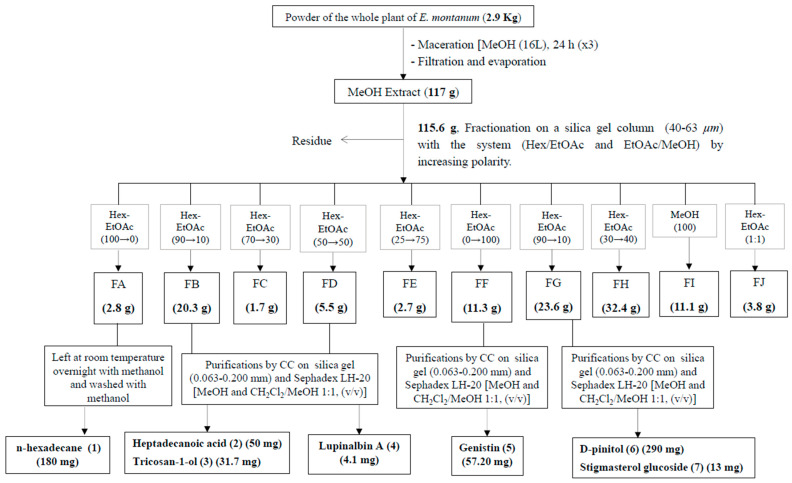
Extraction protocol, fractionation, and isolation of compounds from *E. montanum*.

Figure 1 summarizes the fractionation protocol used for the isolation of compounds. From the MeOH crude extract (117.3 g) of *E. montanum*, part A (115.6 g) was subjected to normal phase silica gel [(40–63 µm, 84.6 g)] column chromatography [CC, length (l) = 600 mm and inner diameter (i.d) = 40 mm], eluted with a mixture of *n*-hexane/EtOAc and EtOAc/MeOH in increasing polarity (100:0, 90:10, 70:30, 50:50, 25:75, 0:100, 90:10, 70:30, 30:70, and 0:100) to yield a total number of 77 fractions of c. 600 mL each. These fractions were further combined into ten main fractions based on their TLC profiling: F_A_ [(1–12), 2.8 g, *n*-hexane/EtOAc (100:0 to 90:10)], F_B_ [(13–18), 20.3 g, *n*-hexane/EtOAc (90:10)], F_C_ [(19–31), 1.7 g, *n*-hexane/EtOAc (90:10)], F_D_ [(32–35), 5.5 g, *n*-hexane/EtOAc (90:10 to 70:30)], F_E_ [(36–44), 2.7 g, *n*-hexane/EtOAc (70:30 to 50:50)], F_F_ [(45–58), 11.3 g, *n*-hexane/EtOAc (50:50 to 0:100)], F_G_ [(59–67), 23.6 g, EtOAc/MeOH (0:100 to 70:30)], F_H_ [(68–72), 32.4 g, EtOAc/MeOH (70:30 to 30:70)], F_I_ [(73–75), 11.1 g, EtOAc/MeOH (30:70 to 0:100)], and F_J_ [(76–77), 3.8 g, EtOAc/MeOH (0:100)].

**Figure 2:**
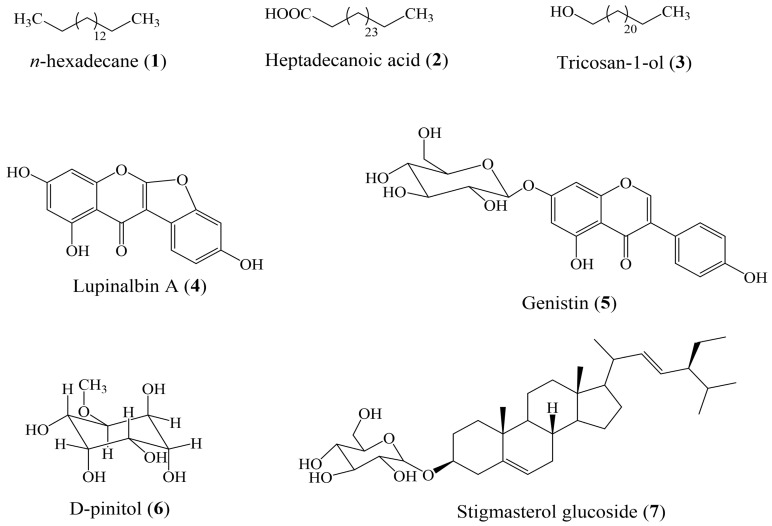
Chemical structures of compounds identified in *E. montanum*.

Fraction F_A_ [(1–12), 2.8 g, *n*-hexane/EtOAc (100:0 to 90:10)] was left at room temperature overnight with methanol; after this operation, crystallization of the powder was observed. The filtration and washing of this white powder several times with methanol yielded compound (**1**) (180 mg). Fraction F_B_ (20 g) was purified via silica gel (40–63 µm, 14.8 g) and CC (l = 425 mm and i.d = 25 mm), eluting with *n*-hexane/EtOAc (100:0, 98:2, 96:4, 94:6, 92:8, 90:10, 87:13, 85:15, 82:18, 79:21, and 75:25) to afford 133 sub-fractions of 40 mL each and pooled into eighteen main sub-fractions: SF_B1 (1)_ [12 mg, *n*-hexane/EtOAc (100:0)], SF_B2 (2–4)_ [16 mg, *n*-hexane/EtOAc (100:0)], SF_B3 (5–10)_ [21 mg, *n*-hexane/EtOAc (98:2)], SF_B4 (11–13)_ [18 mg, *n*-hexane/EtOAc (98:2)], SF_B5 (14)_ [21 mg, *n*-hexane/EtOAc (98:2)], SF_B6 (15–18)_ [41 g, *n*-hexane/EtOAc (98:2)], SF_B7 (19–22)_ [52 mg, *n*-hexane/EtOAc (96:4)], SF_B8 (23–28)_ [35 mg, *n*-hexane/EtOAc (94:6)], SF_B9 (29–37)_ [30 mg, *n*-hexane/EtOAc (92:8)], SF_B10 (38–44)_ [19 mg, *n*-hexane/EtOAc (92:8)], SF_B11 (45–53)_ [22 mg, *n*-hexane/EtOAc (90:10)], SF_B12 (54–60)_ [15 mg, *n*-hexane/EtOAc (90:10)], SF_B13 (61–73)_ [1.7 g, *n*-hexane/EtOAc (87:13)], SF_B14 (74–78)_ [19.5 mg, *n*-hexane/EtOAc (87:13)], SF_B15 (79–101)_ [2.9 g, *n*-hexane/EtOAc (87:13)], SF_B16 (102–111)_ [47 mg, *n*-hexane/EtOAc (85:15)], SF_B17 (113–120)_ [41.5 mg, *n*-hexane/EtOAc (79:21)], and SF_B18 (121–133)_ [7.5 g, *n*-hexane/EtOAc (75:25)]. In the combined sub-fractions of SF_B4 (11–13)_ [18 mg, *n*-hexane/EtOAc (98:2)], a deposit of a white powder was collected, washed with hexane, and submitted to a Sephadex LH-20 chromatographic column (l = 45 mm and i.d = 18 mm) using CH_2_Cl_2_/EtOAc (7:3) to yield (**2**) (50 mg) and (**3**) (31.7 mg). Based on the TLC plate profiling, fractions F_D_ [(32–35), 5.5 g, *n*-hexane/EtOAc (90:10 to 70:30)] and F_E_ [(36–44), 2.7 g, *n*-hexane/EtOAc (70:30 to 50:50)] were mixed and purified by silica gel (40–63 µm, 23.5 g) and CC (l = 425 mm and i.d = 25 mm), eluting with *n*-hexane/acetone (100:0, 95:5, 90:10, 85:15, 80:20, 70:30, 60:40, 1:1, 30:70 and 25:75) to afford 13 sub-fractions of 20 mL each and pooled into four main sub-fractions: SF _(1–2)_ [28 mg, *n*-hexane/acetone (100:0 and 90:10)], SF _(3–6)_ [3.1 g, *n*-hexane/acetone (90:10 to 60:40)], SF _(7–10)_ [1.2 g, *n*-hexane/acetone (1:1)], and SF _(11–13)_ [42 mg, *n*-hexane/acetone (30:70 and 25:75)]. The combined sub-fraction of SF _(11–13)_ [42 mg, *n*-hexane/acetone (30:70 and 25:75)] was purified by Sephadex LH-20 chromatographic column (l = 45 mm and i.d = 18 mm) using CH_2_Cl_2_/MeOH (1:1) to afford 54 sub-fractions; the sub-fraction SF _(11–13) (41–47)_ was subjected again to a Sephadex LH-20 chromatographic column (l = 45 mm and i.d = 18 mm) using CH_2_Cl_2_/MeOH (1:1) to yield (**4**) (4.1 mg). Fraction F_F_ (11.3 g) was purified by silica gel (40–63 µm, 19.7 g) and CC (l = 425 mm and i.d = 25 mm), eluting with *n*-hexane/acetone (100:0, 95:5, 90:10, 85:15, 80:20, 75:25, 70:30, 65:35, 60:40, 50:50 and 0:100) to afford 66 sub-fractions of 50 mL each and pooled into 10 main sub-fractions: SF_1–4_ [16 mg, *n*-hexane/acetone (100:0)], SF_5–19_ [56 mg, *n*-hexane/acetone (100:0 to 95:5)], SF_20–23_ [243 mg, *n*-hexane/acetone (95:5 to 90:10)], SF_24–29_ [1.6 g, *n*-hexane/acetone (90:10 to 85:15)], SF_30_ [350 mg, *n*-hexane/acetone (85:15 to 80:20)], SF_31–34_ [404 mg, *n*-hexane/acetone (80:20 to 75:25)], SF_35–39_ [650 mg, *n*-hexane/acetone (75:25 to 70:30)], SF_40–47_ [2.3 g, *n*-hexane/acetone (70:30 to 65:35)], SF_48–58_ [2.1 g, *n*-hexane/acetone (65:35 to 50:50)], and SF_59–66_ [1.3 g, *n*-hexane/acetone (50:50 to 0:100)]. Combined sub-fraction SF_48–58_ [2.1 g, *n*-hexane/acetone (65:35 to 50:50)] was subjected to a silica gel chromatographic column using an isocratic solvent system [*n*-hexane/acetone (10:90)], followed by a Sephadex LH-20 chromatographic column (l = 45 mm and i.d = 18 mm) using MeOH to afford (**5**) (57.20 mg). Fraction F_G_ [(59–67), 23.6 g, EtOAc/MeOH (0:100 to 70:30)] was left at room temperature overnight with ethyl acetate; after this, a white powder crystallized and was filtrated and washed several times with methanol to yield compound (**6**) (290 mg). The rest of the fraction was purified using silica gel (40–63 µm, 45.6 g) CC (l = 425 mm and i.d = 25 mm), eluting with CH_2_Cl_2_/MeOH (100:0, 95:5, 90:10, 85:15, 80:20, 75:25, 70:30, 65:35, 60:40 and 1:1) to afford 49 sub-fractions of 50 mL each and pooled into 19 main sub-fractions. The sub-fraction SF_G10 (34–35)_ was subjected to a Sephadex LH-20 gel chromatography column using CH_2_Cl_2_/MeOH (1:1) to yield (**7**) (13 mg). Chemical structures of all isolated compounds are presented in Figure 2.

### 2.7. Nuclear Magnetic Resonance (NMR) Spectroscopy for the Characterization of Compounds

The ^1^H and HSQC spectra were acquired using Bruker Avance 400 MHz spectrometer (Ascend TM 400 Bruker, Billerica, MA, USA). The pure compound was prepared and was first stored in a vacuum oven for 4 h at 40 °C to remove any solvent that might be in the sample. Pure sample, 5 mg of the pure compound, was dissolved in 1 mL of a suitable deuterated solvent and then filtered through a Pasteur pipette equipped with a glass wool plug that discharged into a 5 mm NMR tube, which was labeled clearly with a concentric label. The purpose of the filtration was to remove any undissolved sample, particulates, and dust from the solution, which could affect the resolution and the line shape of the NMR spectra. For ^1^H resonances (0 ppm), tetramethylsilane (TMS) was employed as an internal standard. The standard pulse sequences and processing macros given in the original program were used for advanced and two-dimensional spectra such as HSQC. Column chromatography was performed on silica gel Merck 60 F254 [pore size 60 Å, (40–63 µm)] 70–230 and 230–400 mesh, Sigma-Aldrich (Steinheim, Germany), and on Sephadex LH-20 (Sigma-Aldrich, Steinheim, Germany). TLC was performed on silica gel GF/UV_254_ pre-coated plates (Düren, Germany), with detection by spraying with 20% aqueous sulphuric acid (H_2_SO_4_) and heating up to 150 °C.

### 2.8. Determination of IC_50_ Values and Statistical Analysis

The IC_50_ values were calculated for each biological experiment using the nonlinear regression curve of the % inhibitory activity versus the logarithm (log10) of the various tested concentrations using GraphPad Prism 6.0 software (GraphPad Software, Inc., San Diego, CA, USA). The data are shown as mean ⁱ SEM (standard error of the mean) values, and all biological experiments were conducted in triplicate (n = 3). The statistical analysis was conducted using GraphPad Prism 6.0 software (GraphPad Software, Inc., San Diego, CA, USA). One-way analysis of variance (ANOVA) was used to compare the data across the tested samples and/or controls, and Student–Newman–Keuls or Dunnett’s tests were then performed. A *p*-value of less than 0.05 indicated a significant difference in the results.

## 3. Results and Discussion

### 3.1. Phytochemical Composition of E. montanum Methanolic Extract (EMME) and Fractions

**Figure 3:**
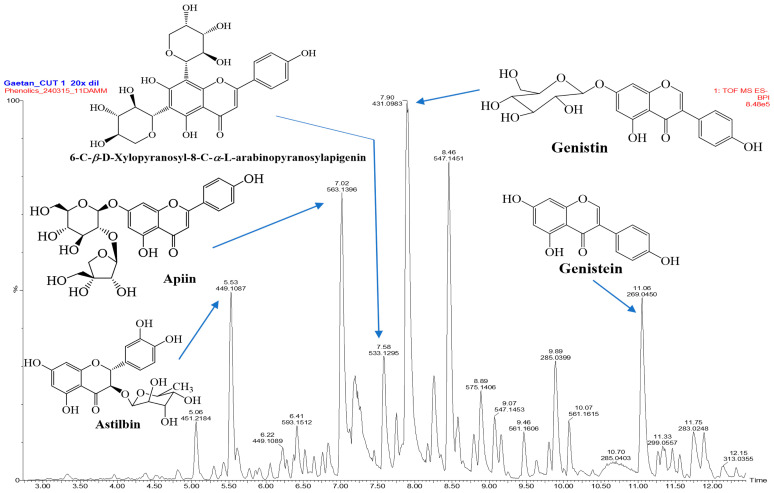
Liquid chromatography–mass spectrometric (LC-MS) profile of *E. montanum* methanolic extract (*EMME*). Major compounds detected: Anopyranosylapigenin (t_R_: 7.585 min), genistin (t_R_: 7.902 min), and genistein (t_R_: 11.055 min).

Two classes of chemicals, flavonoids and phenolics, have been shown to exhibit a number of biological characteristics, such as anti-inflammatory and antioxidant functions, and are crucial in shielding biological systems from the damaging effects of oxidative stress. We measured their amounts in *E. montanum* methanolic extract (EMME), as shown in Table 1, because of their significance for human health. Flavonoids and phenolic compounds were found to be abundant in the EMME extract, with 82.11 mgQE/g and 86.77 mgGAE/g of dry extract, respectively. Since flavonoids and phenolic compounds are known to have strong anti-inflammatory and antioxidant properties, the high concentrations of *EMME* in these phytochemicals imply that they may be involved in *E. montanum*’s anti-inflammatory and antioxidant properties. Astilbin (t_R_: 5.529 min), apiin (t_R_: 7.022 min), 6-C-*β*-d-xylopyranosyl-8-C-*α*-L-arabinopyranosylapigenin (t_R_: 7.585 min), genistin (t_R_: 7.902 min), and genistein (t_R_: 11.055 min) (Figure 3) were the main ingredient found in the LC-MS profile of *EMME*, which also verified the presence of flavonoids and phenolic compounds along with other classes of chemicals such as hydrolyzable tannins, quinic acid derivatives, and lignan glycosides (Table 2).

### 3.2. Antioxidant Activity

Three in vitro tests (ABTS^•+^, DPPH^•^, and FRAP) were used to measure the antioxidant activity of *EMME*. The assay’s ability to quantify the scavenging capacity of radicals was demonstrated by the change in color. The percentage of radicals that were scavenged is reflected in the intensity of the color produced or the degree of discoloration. It is important to note that a higher antioxidant potency is indicated by a lower IC_50_ value. As the positive control in our studies, ascorbic acid, a powerful antioxidant molecule, demonstrated the lowest IC_50_ values against DPPH^•^ and ABTS^•+^ radicals, at 4.08 and 1.28 µg/mL, respectively. Our findings demonstrated that *EMME* has low to moderate ABTS^•+^ and DPPH^•^ radical scavenging potencies, with IC_50_ values of 141.40 µg/mL and 41.28 µg/mL, respectively (Table 2), when compared to ascorbic acid. However, Fractions F_D_, F_E_, and F_F_ demonstrated much greater ABTS^•+^ radical scavenging activity, as seen by their respective IC_50_ values of 23.12, 23.48, and 30.60 µg/mL. With an IC_50_ value of 34.64 µg/mL, fraction F_F_ was the only one to exhibit comparable activity on DPPH^•^. The DPPH^•^ scavenging assay is limited to hydrophobic molecules, whereas the ABTS^•+^ scavenging assay measures both lipophilic and hydrophilic antioxidants [21]. Since DPPH^•^ and ABTS^•+^ assays can be used to examine hydrophilic, hydrophobic, and lipophilic antioxidants, their combination is crucial. Given that antioxidant assays rely on the polarity of active compounds and reaction mechanism, our data suggest that either a different antioxidant mechanism could account for the varying efficacy between ABTS and DPPH assays, or antioxidant compounds within *EMME* or bioactive fractions might have different scavenging mechanisms of action on different radicals [22,23].

### 3.3. Anti-Inflammatory Activity

**Figure 4:**
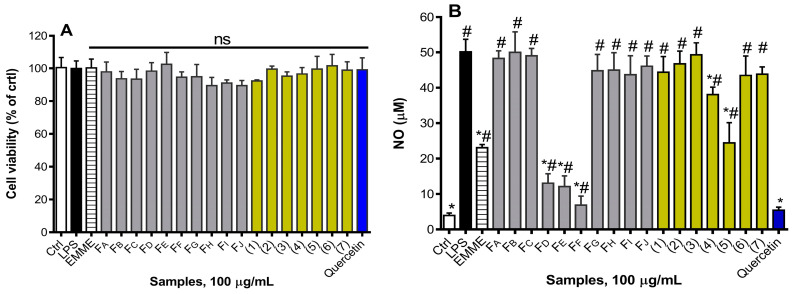
Nitric oxide (NO) production and cell viability in LPS-stimulated RAW 264.7 cells pre-treated with extract, fractions, and purified compounds. (**A**) The cytotoxic effect of tested samples was evaluated using MTT assay; (**B**) RAW 264.7 cells were pre-treated with tested samples at 100 µg/mL for 2 h, followed by exposure to 500 ng/mL of LPS for 24 h to quantify NO in cell supernatants. Each bar depicts the mean ± SD of three replicates (n = 3). One-way ANOVA combined Dunnett or Student–Newman–Keuls’s tests were used for data analysis. * *p* < 0.05 vs. Ctrl. # *p* < 0.05 vs. LPS, ns: non-significant.

**Figure 5:**
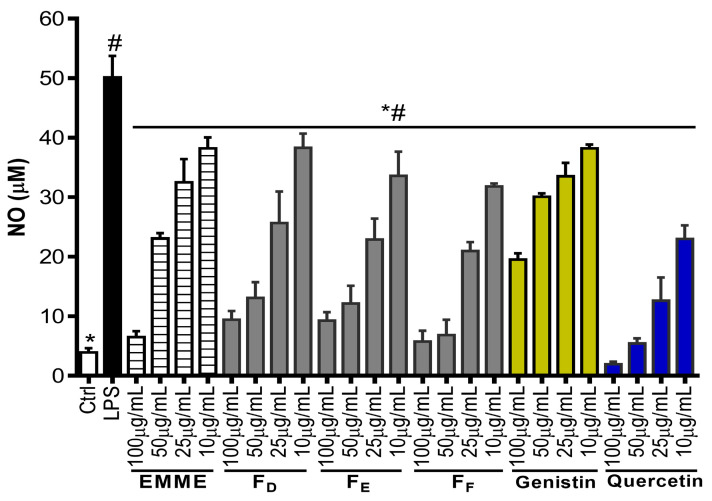
Bioactive samples’ concentration-dependent NO inhibitory action. The means ± SD of duplicate (n = 2) studies are shown for each bar. One-way ANOVA and either Dunnett’s or Student–Newman–Keuls tests were used to evaluate the data. # *p* < 0.01 versus Ctrl. * *p* < 0.01 about LPS.

The NO and 15-LOX inhibitory activities were used to evaluate the anti-inflammatory potential of tested samples. Firstly, EMME, fractions, and isolated compounds were found to be non-significantly cytotoxic to RAW 264.7 cells at 100 µg/mL (Figure 4A). Further, it was observed that at 100 µg/mL, EMME and fractions F_D_, F_E_, and F_F_, as well as compound (**5**), significantly inhibited LPS-induced NO production as compared to control cells (Figure 4B). The dose-dependent effect of bioactive samples showed that F_F_ highly inhibited NO production, with an IC_50_ value of 16.43 µg/mL compared to 35.78 µg/mL for EMME (Figure 5). Similarly, EMME showed low efficacy against 15-LOX, with an IC_50_ value of 226.20 µg/mL, while fraction F_F_ exhibited improved efficacy, with an IC_50_ value of 77.29 µg/mL (Table 2). These data suggest that bioactive compounds responsible for this bioactivity might be more concentrated in this fraction. Thus, compound (**5**), identified as genistin and isolated from fraction F_F_, was the only compound that efficiently inhibited NO production, with an IC_50_ value of 66.01 µg/mL. Additionally, genistin moderately scavenges ABTS and DPPH free radicals, with IC_50_ values of 99.89 and 91.02 µg/mL, respectively (Table 3). Genistin was also able to inhibit 15-LOX in a dose-dependent manner, with an IC_50_ value of 179.00 µg/mL. As genistin was less active compared to fraction F_F_, it therefore means that other compounds within this fraction might interact synergistically with genistin to yield its high antioxidant and anti-inflammatory potential.

### 3.4. Effect of E. montanum Methanolic Extract and Genistin on the Production of Reactive Oxygen Species

**Figure 6:**
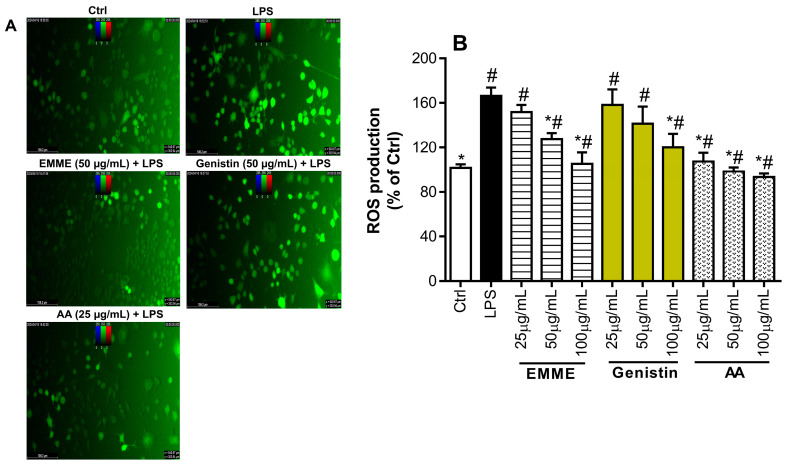
Reactive oxygen species (ROS) production in LPS-stimulated RAW 264.7 cells. RAW 264.7 cells were pre-treated with different concentrations (25, 50, and 100 µg/mL) of *E. montanum* methanolic extract (*EMME*), genistin, and ascorbic acid (AA) for two hours, then exposed to 200 ng/mL of LPS for twenty-four hours. Cell fluorescence was monitored at 485 nm (excitation) and 535 nm (emission) (**A**), and intracellular ROS levels were assessed using the DCFH-DA probe (10 µM). Percentages of negative control cells (**B**) are used to represent intracellular ROS levels. The means ± SD of three studies in triplicate are shown by each bar. One-way ANOVA and either Dunnett’s or Student–Newman–Keuls tests were used to evaluate the data. * *p* < 0.05 vs. LPS, # *p* < 0.05 vs. Ctrl.

We assessed the protective effects of *EMME* and genistin against LPS-induced oxidative stress in RAW 264.7 murine macrophages by measuring ROS levels using DCFH-DA in order to better understand their anti-inflammatory and antioxidant mechanisms of action. The protective effect of *EMME* and genistin against ROS generation is depicted in Figure 6. When compared to untreated cells (Ctrl), ROS generation was significantly increased when RAW 264.7 cells were treated with LPS (Figure 6A). LPS-induced ROS generation was markedly and dose-dependently reduced when RAW 264.7 murine macrophages were pre-treated with *EMME* or genistin (Figure 6B). Ascorbic acid, which was employed as a positive control, was found to significantly inhibit the generation of ROS generated by LPS, but *EMME* was found to be more effective than genistin. Ascorbic acid is a well-known antioxidant and anti-inflammatory substance that can scavenge radicals and prevent the generation of reactive oxygen species (ROS) by activating intracellular antioxidant mechanisms. Our findings indicated that *EMME*’s antioxidant potential could lower the generation of intracellular ROS that were triggered by LPS. Additionally, it demonstrated its anti-inflammatory and antioxidant capabilities by shielding RAW 264.7 cells from the harmful effects of ROS. Although genistin is the main component of *EMME*, its modest effectiveness in reducing ROS production in comparison to *EMME* suggests that it interacts with other *EMME* chemicals that were inferred from LC-MS analysis.

### 3.5. LC-MS Profile of E. montanum MeOH Extract (EMME)

The LC-MS profile of the *E. montanum* methanolic extract (EMME) showed genistin [961.99 mg/L (Conc. in extract vs. Catechin (mg/L)], one of the most commonly found isoflavonoid glycosides in different parts of many plant species that is commonly produced by plant metabolism and detected in several plant species, particularly in the Fabaceae family. Genistin has been reported to have moderate antioxidant properties via its scavenging activity against peroxynitrite (ONOO^−^), DPPH^•^, and ABTS^•+^ radicals [24,25]. Additionally, other studies revealed that genistin decreases LPS-induced NO production and suppresses the expression levels of pro-inflammatory cytokines (IL-6, IL-8, and TNF-α) by blocking the P2X7/NF-kβ pathways [26,27,28]. Our results support these previous data and confirm the antioxidant and anti-inflammatory potentials of genistin, which is usually metabolized into genistein in the gastrointestinal tract by intestinal bacteria. Other compounds were also identified in EMME, and this includes genistein [428.60 mg/L (Conc. in extract vs. Catechin (mg/L)], which possessed strong anti-inflammatory activities by down-regulating the expression of prostaglandins, inducible nitric oxide synthase (iNOS), pro-inflammatory cytokines, reactive oxygen species (ROS), and the NF-κB signaling pathway [29,30]. Astilbin [448.27 mg/L (Conc. in extract vs. Catechin (mg/L)] also demonstrated anti-inflammatory properties by significantly inhibiting the production of IL-1β, IL-6, NO, and the expression of NF-κB-p65 in LPS-stimulated RAW264.7 cells [31,32]. Apiin [619.38 mg/L (Conc. in extract vs. Catechin (mg/L)] showed significant inhibitory activity on NO production and iNOS expression in LPS-activated J774.A1 cells [33]. Taken together, the identification of these compounds in significant amounts in *EMME* supports their substantial contribution to the anti-inflammatory and antioxidant properties of *E. montanum.* These data justify the medicinal use of this plant species against oxidative stress-related illnesses, thus making *E. montanum* a source of antioxidant and inflammatory agents. Further exploration of *E. montanum* is required to fully characterize several unidentified chemicals that could provide new insights into the isolation of more bioactive compounds in *E. montanum*, a plant species that is phytochemically under-explored to date.

### 3.6. Chemical Identification of Isolated Compounds

The chemical structures of isolated compounds were elucidated based on their NMR spectroscopic data (Figures S1–S16, Supplementary Materials). These data were compared with the available literature, and they were identified as *n*-hexadecane (**1**) [34], heptacosanoic acid (**2**) [35], tricosan-1-ol (**3**) [36], lupinalbin A (**4**) [4,37,38], genistin (**5**) [4,39], d-pinitol (**6**) [4], and stigmasterol glucoside (**7**) [4,40]. Regarding LC-MS profiling, although 6-C-*β*-d-xylopyranosyl-8-C-*α*-L-arabinopyranosylapigenin, apiin, and astilbin are also part of the major ones of this plant, they have not yet been either fully characterized or isolated. It is important to note that another compound found in a relatively high concentration (RT about 8.5 min) was reported in the table as an unknown compound.

### 3.7. Chemotaxonomic Significance

Plant secondary metabolites are rich sources of bioactive compounds eliciting many beneficial human health effects. Plant-based foods, including vegetables, fruits, seeds, and legumes, may contain hundreds of different phytochemicals. This is the case of an African medicinal plant, namely, *Eriosema montanum* (Fabaceae,) from which twenty-five known compounds have already been isolated and characterized. In the current study, three identified compounds, namely, *n*-hexadecane (**1**), heptacosanoic acid (**2**), and tricosan-1-ol (**3**), were isolated for the first time from the whole plant of *E. montanum* methanolic extract. Furthermore, the previous phytochemical analyses conducted on the CH_2_Cl_2_-MeOH (1:1) extract of the roots, stem bark, and leaves of *E. montanum* have led to the isolation of lupinalbin A (**4**), genistin (**5**), d-pinitol (**6**), stigmasterol glucoside (**7**), genistein, malonyl genistin, isoprunetin, isoluteolin, salicylic acid, 4-hydroxy-5,5-dimethyldihydrofuran-2-one, *p*-coumaric acid, eucomic acid, propanoic acid, montachalcone A, montachalcone B, 2′,4′,5,6′-tetrahydroxy-4-methoxy-3,3′-diprenyldihydrochalcone, 2′,4′,4,6′-tetrahydroxy-3,3′-diprenyldihydrochalcone, isorhamnetin, stigmasterol, cedrol, 4-hydroxy-5,5-dimethyldihydrofuran-2-one, betulinic acid, and dodecyl-3-(3,4-dihydroxyphenyl)propenoate [3,4]. Genistin (**5**) was previously isolated from the CH_2_Cl_2_-MeOH (1:1) extract of the roots of *E. montanum* [3,4], *E. glomeratum* [5], *E. Laurentii* [41,42], *E. chinense* [43], and *E. tuberosum* [44]. Compounds (**4**) and (**7**) were previously isolated from the CH_2_Cl_2_-MeOH (1:1) extract of the leaves of *E. montanum* [4], while compound (**6**) was isolated from the stem bark of the CH_2_Cl_2_-MeOH (1:1) of *E. montanum* [4]. Further, compound (**3**) was previously isolated from the CH_2_Cl_2_-MeOH (1:1) extract of the aerial part of *E. glomeratum* [4], and montachalcone A and B were characterized for the first time in *E. montanum* [4], whereas isoluteolin was isolated from the whole plant of *E. glomeratum* [45]. Genistein was previously isolated from the roots and twigs of *E. tuberosum* [7,44], the roots of *E. chinense* [43], and the whole plant part of *E. Laurentii* [41,42]. In order to fully understand the chemotaxonomy significance of *Eriosema montanum*, its isolated compounds are summarized in Table 4. By comparing the data from this plant species and available literature, in total, twenty-seven plant metabolites were isolated from the different parts of *Eriosema montanum*, as well as four other different species, and these include six isoflavonoids, five phenol acids, four dihydrochalcones, three flavones, two steroids, one sesquiterpene, one sugar, one lactone, one pentacyclic triterpene, one flavonol, one alkyl caffeate derivative, one alcohol, one carboxylic acid, and one alkane. The literature review allowed us to collect five different species where the compounds were also isolated, all belonging to the Fabaceae family. This result provides additional information on the chemical characteristics of the Fabaceae family. Some flavonoids, as well as genistein, genistin, malonyl genistin, isoprunetin, 7-*O*-glucopyranosyl-isoprunetin, isoluteolin, and quercetin, have been mostly isolated in the same part (the roots) of *E. montanum*, as well as many other species [4,5], and this allows us to suggest that many classes of flavonoids (isoflavonoids and flavonol) are detected in the roots of Eriosema genus. On the other hand, the isolation of dihydrochalcones such as montachalcone A, montachalcone B, 2′,4′,5,6′-tetrahydroxy-4-methoxy-3,3′-diprenyldihydrochalcone, and 2′,4′,4,6′-tetrahydroxy-3,3′-diprenyldihydrochalcone in the leaves of *E. montanum* [4] might suggest that this particular class of flavonoid (dihydrochalcone) is mostly detected in the leaves of Eriosema genus. Moreover, previous research on *Eriosema* plant species revealed the presence of dihydrochalcones, flavonoids, isoflavonoids, terpenoids, triterpenoids, fatty acids, and alcohols [5,6,41,42,43,45]. Given our results obtained on the isolation of lupinalbin A (**4**) and genistin (**5**) from the methanolic extract of *E. montanum* compared to previous studies, isoflavonoids and flavones have chemotaxonomic importance within each identified species.

## 4. Conclusions

The further phytochemical investigation of *E. montanum* methanolic extract (EMME) afforded seven known natural compounds (**1**–**7**). The LC-MS allowed us, for the first time, to establish the tentative identification of phytoconstituents, among which genistin (**5**) was detected as the major compound of *E. montanum*. The antioxidant and anti-inflammatory activities of EMME were attributed to the presence of genistin and other compounds that have been pharmacologically recognized as known antioxidant and anti-inflammatory agents. Genistin (**5**), isolated from EMME, exhibited moderate radical scavenging potential, but it was able to inhibit LPS-induced NO and ROS production in RAW 264.7 macrophages. Most importantly, EMME, isolated fractions, and compounds were non-toxic to these cells up to 100 µg/mL. However, genistin was less effective than Fraction F_F_ from which it was isolated, thus suggesting that other bioactive compounds might interact synergistically to support the higher efficacy of this fraction, as well as the crude extract EMME. Our results support the health benefits of *E. montanum* for managing oxidative-stress-related diseases, and this study opens further perspectives to explore the minor constituents of this plant, as this might result in the discovery of novel compounds.

## Figures and Tables

**Table 1:** Phytochemical contents and antioxidant and anti-inflammatory activities of crude extract and its main fractions.

Tested Samples	TPC (mg GAE/g of Extract)	TFC (mg QE/g of Extract)	IC_50_ (µg/mL)				
			DPPH	ABTS	FRAP	NO	15-LOX
**Extract**	86.77 ± 6.24	82.11 ± 4.60	141.40 ± 4.66	41.28 ± 1.03	>200	35.78 ± 4.12	226.20 ± 3.32
**F_A_**	2.87 ± 0.11	10.66 ± 0.52	>500	>500	>200	>100	>500
**F_B_**	21.86 ± 6.22	52.69 ± 1.01	>500	930.90 ± 2.13	>200	>100	>500
**F_C_**	60.38 ± 1.06	67.16 ± 5.91	859.30 ± 2.78	164.00 ± 1.46	>200	>100	>500
**F_D_**	105.69 ± 0.29	84.84 ± 3.30	142.30 ± 1.08	23.12 ± 1.07	>200	25.14 ± 1.35	150.60 ± 1.53
**F_E_**	115.95 ± 1.51	59.82 ± 8.47	156.60 ± 5.63	23.48 ± 1.23	>200	19.85 ± 1.23	195.20 ± 2.57
**F_F_**	86.82 ± 0.81	85.70 ± 6.00	34.64 ± 1.75	30.60 ± 1.22	>200	16.43 ± 1.76	77.29 ± 2.11
**F_G_**	76.29 ± 1.07	59.58 ± 2.11	185.40 ± 1.10	73.97 ± 1.53	>200	>100	>500
**F_H_**	81.86 ± 2.68	48.31 ± 2.61	126.20 ± 1.94	53.61 ± 1.61	>200	>100	>500
**F_I_**	63.84 ± 0.93	16.01 ± 2.30	232.80 ± 1.83	88.85 ± 1.52	>200	>100	>500
**F_J_**	44.72 ± 2.46	4.75 ± 0.47	669.40 ± 5.48	98.53 ± 1.55	>200	>100	>500
**Ascorbic acid**	N/A	N/A	4.08 ± 1.04	1.28 ± 0.81	23.68 ± 2.89	N/A	N/A
**Quercetin**	N/A	N/A	N/A	N/A	N/A	8.93 ± 0.40	N/A
**Gallic acid**	N/A	N/A	N/A	N/A		N/A	24.73 ± 2.08

TPC: total phenolic content; TFC: total flavonoid content; GAE: gallic acid equivalents; QE: quercetin equivalents; N/A: Not-Applicable.

**Table 2:** Phytochemical compounds detected and characterized in *E. montanum* methanolic extract of the whole plant using liquid chromatography–mass spectrometric in negative mode ionization.

Peak No.	t_R_ (min)	[M-H] (*m*/*z*)	Tentative Assignment (Compound Names)	Ontology	Molecular Formula	Total Score	Peak Height Intensity	Conc. in Extract vs. Catechin (mg/L)
1	4.118	315.0717	Gentesic acid 5-*O*-glucoside	Phenolic glycosides	C_13_H_16_O_9_	7.3147	3438	3.652952
2	4.343	153.0192	3,5-Dihydroxybenzoic acid	Hydroxybenzoic acid derivatives	C_7_H_6_O_4_	8.1282	1804	1.687143
3	4.829	359.0979	Glucosyringic acid	Hydrolyzable tannins	C_15_H_20_O_10_	6.4985	16,201	19.00768
4	5.056	451.2184	3-*O*-*β*-glucopyranosyl-(1→6)-*β*-glucopyranosyl-1-octen-3-ol	Fatty acyl glycosides of mono- and disaccharides	C_20_H_36_O_11_	5.722	112,039	134.3071
5	5.386	367.1606	(2*S*)-2-Butanol *O*-[b-d-Apiofuranosyl-(1→6)-b-d-glucopyranoside]	O-glycosyl compounds	C_15_H_28_O_10_	4.2327	9685	11.1685
6	5.529	449.1087	**Astilbin**	Flavonoid-3-O-glycosides	C_21_H_22_O_11_	7.8065	373,009	448.271
7	5.587	325.0932	1-O-*p*-Coumaroyl-*β*-d-glucose	Hydroxycinnamic acid glycosides	C_15_H_18_O_8_	6.5163	19,055	22.44122
8	6.198	423.0939	3-Glucosyl-2,3′,4,4′,6-pentahydroxybenzophenone	Phenolic glycosides	C_19_H_20_O_11_	7.0265	956	0.666943
9	6.27	401.1451	Benzyl *β*-primeveroside	O-glycosyl compounds	C_18_H_26_O_10_	6.5452	33,557	39.88808
10	6.415	593.1513	Neosaponarin	Flavonoid-7-O-glycosides	C_27_H_30_O_15_	7.4247	106,481	127.6204
11	6.747	431.1921	Citroside A	Terpene glycosides	C_19_H_30_O_8_	5.8817	53,354	63.70516
12	7.022	563.1399	**Apiin**	Flavonoid-7-O-glycosides	C_26_H_28_O_14_	7.8008	515,238	619.3818
13	7.197	447.0931	Luteolin 7-glucoside	Flavonoid-7-O-glycosides	C_21_H_20_O_11_	7.7413	221,470	265.9597
14	7.229	581.2242	(7′R)-(+)-Lyoniresinol 9′-glucoside	Lignan glycosides	C_28_H_38_O_13_	7.5392	770	0.443173
15	7.53	475.1832	Phenethyl rutinoside	O-glycosyl compounds	C_20_H_30_O_10_	5.2453	5355	5.959228
16	7.585	533.1295	**6-C-*β*-d-Xylopyranosyl-8-C-*α*-L-arabinopyranosylapigenin**	Flavonoid 8-C-glycosides	C_25_H_26_O_13_	7.1136	252,385	303.1525
17	7.709	433.1149	5-hydroxy-2-(4-hydroxyphenyl)-7-{[3,4,5-trihydroxy-6-(hydroxymethyl)oxan-2-yl]oxy}-3,4-dihydro-*2H*-1-benzopyran-4-one	Flavonoid-7-O-glycosides	C_21_H_22_O_10_	7.5518	5009	5.542967
18	7.754	577.1562	Cassitoroside	Phenolic glycosides	C_25_H_32_O_14_	6.9928	129,446	155.2488
19	7.763	463.1246	Hesperetin 7-glucoside	Flavonoid-7-O-glycosides	C_22_H_24_O_11_	7.785	768	0.440767
20	7.902	431.0981	**Genistin**	Isoflavonoid O-glycosides	C_21_H_20_O_10_	8.6105	800,025	961.9992
21	8.253	477.1035	Cosmosiin	Flavonoid-7-O-glycosides	C_21_H_20_O_10_	7.5523	215,138	258.3419
22	8.585	503.1772	(*S*)-Multifidol 2-[apiosyl-(1→6)-glucoside]	Phenolic glycosides	C_22_H_32_O_13_	6.0192	121,523	145.7169
23	8.888	597.1226	Maysin	Flavonoid C-glycosides	C_27_H_28_O_14_	7.5346	24,980	29.56939
24	8.986	415.1971	Ethyl 7-epi-12-hydroxyjasmonate glucoside	Fatty acyl glycosides of mono- and disaccharides	C_20_H_32_O_9_	5.8442	6713	7.592991
25	9.078	547.1448	Mirificin	Isoflavonoid C-glycosides	C_26_H_28_O_13_	6.9171	125,297	150.2573
26	9.08	341.1032	Dulxanthone D	8-prenylated xanthones	C_19_H_18_O_6_	6.6051	1181	0.937633
27	9.162	371.1342	Syringenone	Iridoid O-glycosides	C_17_H_24_O_9_	6.4187	91,380	109.4529
28	9.19	405.1196	Afzelechin 7-apioside	Flavan-3-ols	C_20_H_22_O_9_	7.327	2625	2.67486
29	9.327	461.2399	Neryl rhamnosyl-glucoside	Terpene glycosides	C_22_H_38_O_10_	7.1274	1886	1.785794
30	9.465	561.1606	Molludistin 2″-rhamnoside	Flavonoid 8-C-glycosides	C_27_H_30_O_13_	6.9709	91,264	109.3134
31	9.633	283.061	Biochanin A	4′-O-methylisoflavones	C_16_H_12_O_5_	7.1595	40,552	48.30352
32	9.796	463.2545	Pumilaside A	Terpene glycosides	C_21_H_38_O_8_	5.8818	29,146	34.58136
33	9.802	581.187	10-Acetoxyligustroside	Terpene glycosides	C_27_H_34_O_14_	7.0346	73,861	88.37643
34	9.901	745.2705	Alangisesquin A	2-arylbenzofuran flavonoids	C_37_H_46_O_16_	6.4322	20,045	23.63226
35	10.155	461.2397	Xi-Linalool 3-[rhamnosyl-(1→6)-glucoside]	Fatty acyl glycosides of mono- and disaccharides	C_22_H_38_O_10_	5.7354	24,176	28.60212
36	10.332	461.2388	Neryl rhamnosyl-glucoside	Terpene glycosides	C_22_H_38_O_10_	5.7676	8258	9.451727
37	10.379	461.1088	Diosmetin 7-*O*-*β*-d-glucopyranoside	Flavonoid-7-O-glycosides	C_22_H_22_O_11_	7.394	6267	7.056424
38	11.055	269.045	**Genistein**	Isoflavones	C_15_H_10_O_5_	8.5921	356,661	428.6033
39	11.248	583.2187	Propinquanin E	Hydrolyzable tannins	C_31_H_36_O_11_	6.7249	11,228	13.02483
40	11.267	927.4969	Araliasaponin I	Triterpene saponins	C_47_H_76_O_18_	6.9407	57,168	68.29366
41	11.337	299.0559	Chrysoeriol	3′-O-methylated flavonoids	C_16_H_12_O_6_	7.9788	69,005	82.53434
42	11.456	327.2174	Corchorifatty acid F	Lineolic acids and derivatives	C_18_H_32_O_5_	6.0863	61,804	73.87107
43	11.563	433.1502	Vestitone 7-glucoside	Isoflavonoid O-glycosides	C_22_H_26_O_9_	6.7595	49,565	59.14675
44	11.884	329.2332	9,12,13-TriHOME	Long-chain fatty acids	C_18_H_34_O_5_	6.0998	95,405	114.2953
45	12.14	313.0353	Laccaic acid D	Anthracenecarboxylic acids	C_16_H_10_O_7_	6.9492	30,465	36.1682
46	12.183	925.4796	Araloside A	Triterpene saponins	C_47_H_74_O_18_	6.5655	686	0.342116
47	12.32	939.4968	Dehydrosoyasaponin I	Triterpene saponins	C_48_H_76_O_18_	6.5386	24,212	28.64543
48	12.464	385.1298	Topazolin hydrate; or 5,7-Dihydroxy-6-(3-hydroxy-3-methylbutyl)-2-(4-hydroxyphenyl)-3-methoxy-*4H*-1-benzopyran-4-one	6-prenylated flavones	C_21_H_22_O_7_	6.4238	16,145	18.9403
49	12.626	263.129	(S)-Abscisic acid	Abscisic acids and derivatives	C_15_H_20_O_4_	7.4057	66,665	79.71917
50	12.94	311.1862	(+)-Neomethynolide; Neomethynolide	Macrolides and analogues	C_17_H_28_O_5_	4.3637	3053	3.189772
51	5.587	315.0717	Gentesic acid 5-*O*-glucoside	Phenolic glycosides	C_13_H_16_O_9_	7.3147	3438	3.652952

t_R_: retention time.

**Table 3:** Antioxidant and anti-inflammatory activities of isolated compounds.

Compounds	IC_50_ (µg/mL)				
	DPPH	ABTS	FRAP	NO	15-LOX
**1**	>100	>100	>200	>100	>200
**2**	>100	>100	>200	>100	>200
**3**	>100	>100	>200	>100	>200
**4**	>100	>100	>200	>100	>200
**5**	99.89 ± 1.26	91.02 ± 1.33	>200	66.01 ± 4.21	179.00 ± 2.24
**6**	>100	>100	>200	>100	>200
**7**	>100	>100	>200	>100	>200
Ascorbic acid	4.08 ± 1.04	1.28 ± 0.81	23.68 ± 2.89	N/A	N/A
Quercetin	N/A	N/A	N/A	8.93 ± 0.40	N/A
Gallic acid	N/A	N/A	N/A	N/A	24.73 ± 2.08

N/A: Not-Applicable.

**Table 4:** Isolated compounds from *Eriosema montanum* (Fabaceae) found in the other species.

Compound Names	*E. montanum*			*E. glomeratum*			*E. tuberosum*			*E. chinense*			*E. laurentii*		
	L	R	Sb	L	R	Sb	L	R	Sb	L	R	Sb	L	R	Sb
* *n*-Hexadecane (**1**)	**+**ns	**+**ns	**+**ns	**-**	**-**	**-**	**-**	**-**	**-**	**-**	**-**	**-**	**-**	**-**	**-**
* Heptacosanoic acid (**2**)	**+**ns	**+**ns	**+**ns	**-**	**-**	**-**	**-**	**-**	**-**	**-**	**-**	**-**	**-**	**-**	**-**
* Tricosan-1-ol (**3**)	**+**ns	**+**ns	**+**ns	**-**	**-**	**-**	**-**	**-**	**-**	**-**	**-**	**-**	**-**	**-**	**-**
* Lupinalbin A (**4**)	**+**	**-**	**-**	**+**	**-**	**-**	**-**	**-**	**-**	**-**	**-**	**-**	**-**	**-**	**-**
* Genistin (**5**)	**-**	**+**	**-**	**-**	**+**	**-**	**-**	**+**	**-**	**-**	**+**	**-**	**+**	**-**	**-**
* d-pinitol (**6**)	**-**	**-**	**+**	**-**	**-**	**-**	**-**	**-**	**-**	**-**	**-**	**-**	**-**	**-**	**-**
* Stigmasterol glucoside (**7**)	**+**	**-**	**-**	**-**	**-**	**-**	**-**	**-**	**-**	**-**	**-**	**-**	**-**	**-**	**-**
Genistein	**-**	**+**	**-**	**-**	**-**	**-**	**-**	**+**	**-**	**-**	**-**	**-**	**+**	**-**	**-**
Malonyl genistin	**-**	**+**	**-**	**-**	**-**	**-**	**-**	**-**	**-**	**-**	**-**	**-**	**-**	**-**	**-**
Isoprunetin	**-**	**+**	**-**	**-**	**-**	**-**	**-**	**-**	**-**	**-**	**-**	**-**	**-**	**-**	**-**
7-*O*-Glucopyranosyl-isoprunetin	**-**	**+**	**-**	**-**	**-**	**-**	**-**	**-**	**-**	**-**	**-**	**-**	**-**	**-**	**-**
Isoluteolin	**-**	**+**	**-**	**+**	**-**	**-**	**-**	**-**	**-**	**-**	**-**	**-**	**-**	**-**	**-**
Salicylic acid	**-**	**-**	**+**	**-**	**-**	**-**	**-**	**-**	**-**	**-**	**-**	**-**	**-**	**-**	**-**
3-(4-Methoxyphenyl)propanoic acid	**-**	**+**	**-**	**-**	**-**	**-**	**-**	**-**	**-**	**-**	**-**	**-**	**-**	**-**	**-**
*p*-Coumaric acid	**-**	**-**	**+**	**-**	**-**	**-**	**-**	**-**	**-**	**-**	**-**	**-**	**-**	**-**	**-**
Eucomic acid	**-**	**-**	**+**	**-**	**-**	**-**	**-**	**-**	**-**	**-**	**-**	**-**	**-**	**-**	**-**
Montachalcone A	**+**	**-**	**-**	**-**	**-**	**-**	**-**	**-**	**-**	**-**	**-**	**-**	**-**	**-**	**-**
Montachalcone B	**+**	**-**	**-**	**-**	**-**	**-**	**-**	**-**	**-**	**-**	**-**	**-**	**-**	**-**	**-**
2′,4′,5,6′-Tetrahydroxy-4-methoxy-3,3′-Diprenyldihydrochalcone	**+**	**-**	**-**	**-**	**-**	**-**	**-**	**-**	**-**	**-**	**-**	**-**	**-**	**-**	**-**
2′,4′,4,6′-Tetrahydroxy-3,3′-diprenyldihydrochalcone	**+**	**-**	**-**	**-**	**-**	**-**	**-**	**-**	**-**	**-**	**-**	**-**	**-**	**-**	**-**
Isorhamnetin	**-**	**+**	**-**	**-**	**-**	**-**	**-**	**-**	**-**	**-**	**-**	**-**	**-**	**-**	**-**
Quercetin	**-**	**+**	**-**	**-**	**-**	**-**	**-**	**-**	**-**	**-**	**-**	**-**	**-**	**-**	**-**
Stigmasterol	**+**	**+**	**-**	**-**	**-**	**-**	**-**	**-**	**-**	**-**	**-**	**-**	**-**	**-**	**-**
Cedrol	**-**	**+**	**-**	**-**	**-**	**-**	**-**	**-**	**-**	**-**	**-**	**-**	**-**	**-**	**-**
4-Hydroxy-5,5-dimethyldihydrofuran-2-one	**+**	**-**	**-**	**-**	**-**	**-**	**-**	**-**	**-**	**-**	**-**	**-**	**-**	**-**	**-**
Betulinic acid	**+**	**-**	**-**	**-**	**-**	**-**	**-**	**-**	**-**	**-**	**-**	**-**	**-**	**-**	**-**
Dodecyl-3-(3,4- dihydroxyphenyl)propenoate	**+**	**-**	**-**	**-**	**-**	**-**	**-**	**-**	**-**	**-**	**-**	**-**	**-**	**-**	**-**

L: leaves; R: roots; Sb: stem Bark; (+): present; (-): not available; ns: non-specified *: compounds isolated in this work from the methanolic extract of the whole plant of the Cameroonian species of *E. montanum*.

## Data Availability

The datasets used and/or analyzed during the current study are available from the corresponding authors upon reasonable request.

## Supplementary Materials

The following supporting information can be downloaded at: https://www.mdpi.com/article/10.3390/molecules29245885/s1, Figure S1: ^1^H NMR (400 MHz) spectrum of compound 1 recorded in CDCl_3_; Figure S2: ^13^C NMR (100 MHz) spectrum of compound 1 recorded in CDCl_3_; Figure S3: ^1^H NMR (400 MHz) spectrum of compound 2 recorded in CDCl_3_; Figure S4: ^1^H NMR (400 MHz) spectrum of compound 3 recorded in CDCl_3_; Figure S5: ^1^H NMR (600 MHz) spectrum of compound 4 recorded in DMSO-*d_6_*; Figure S6: ^13^C NMR (150 MHz) spectrum of compound 4 recorded in DMSO-*d_6_*; Figure S7: HSQC spectrum of compound 4 recorded in DMSO-*d_6_*; Figure S8: COSY spectrum of compound 4 recorded in DMSO-*d_6_*; Figure S9: ^1^H NMR (600 MHz) spectrum of compound 5 recorded in DMSO-*d_6_*; Figure S10: ^13^C NMR (150 MHz) spectrum of 5 in DMSO-*d_6_*; Figure S11: HSQC spectrum of compound 5 recorded in DMSO-*d_6_*; Figure S12: ^1^H NMR (600 MHz) spectrum of compound 6 recorded in CD_3_OD; Figure S13: ^13^C NMR (150 MHz) spectrum of compound 6 recorded in CD_3_OD; Figure S14: HSQC spectrum of compound 6 recorded in CD_3_OD; Figure S15: ^1^H NMR (600 MHz) spectrum of compound 7 recorded in DMSO-*d_6_*; Figure S16: ^13^C NMR (150 MHz) spectrum of compound 7 recorded in DMSO-*d_6_*. Figure S17: Calibration curve of pick intensity vs. concentration of catechin.
