# Supplementary material for: The Antioxidant and Anti-Inflammatory Activities of the Methanolic Extract, Fractions, and Isolated Compounds from Eriosema montanum Baker f. (Fabaceae)
Source: Molecules. 2024 Dec 13;29(24):5885. doi: 10.3390/molecules29245885 (PMC11678782; doi:10.3390/molecules29245885)
Supplement: Supplementary file 1 [file molecules-29-05885-s001.zip › molecules-3357457-supplementary.pdf]

SUPPLEMENTARY MATERIAL

Antioxidant and anti-inflammatory activities of the methanolic extract, fractions and isolated compounds from *Eriosema montanum* Baker f. (Fabaceae).

Gaétan Tchangou Tabakam [1,2], Emmanuel Mfotie Njoya [1], Chika Ifeanyi Chukwuma [1], Samson Sitheni Mashele [1], Yves Martial Mba Nguekeu [2], Mathieu Tene [2], Maurice Ducret Awouafack [2,*] and Tshepiso Jan Makhafola [1,*]

[1]*Centre for Quality of Health and Living, Faculty of Health and Environmental Sciences, Central University of Technology, Bloemfontein 9300, Free State, South Africa;*

[2]*Natural Products Chemistry Research Unit, Department of Chemistry, Faculty of Science, University of Dschang, P.O. Box 67, Dschang, Cameroon.*

*Correspondences: ducret.awouafack@univ-dschang.org (M.D. Awouafack) and jmakhafola@cut.ac.za (T.J. Makhafola).

List of supplementary content

No.	Contents	Page
1	**Figure S1.** ^{1}H NMR (400 MHz) spectrum of compound **1** recorded in $CDCl_3$	3
2	**Figure S2.** ^{13}C NMR (100 MHz) spectrum of compound **1** recorded in $CDCl_3$	3
3	**Figure S3.** ^{1}H NMR (400 MHz) spectrum of compound **2** recorded in $CDCl_3$	4
4	**Figure S4.** ^{1}H NMR (400 MHz) spectrum of compound **3** recorded in $CDCl_3$	4
5	**Figure S5.** ^{1}H NMR (600 MHz) spectrum of compound **4** recorded in DMSO-d_6	5
6	**Figure S6.** ^{13}C NMR (150 MHz) spectrum of ocmpound **4** recorded in DMSO-d_6	5
7	**Figure S7.** HSQC spectrum of compound **4** recorded in DMSO-d_6	6
8	**Figure S8.** COSY spectrum of compound **4** recorded in DMSO-d_6	6
9	**Figure S9.** ^{1}H NMR (600 MHz) spectrum of compund **5** recorded in DMSO-d_6	7
10	**Figure S10.** ^{13}C NMR (150 MHz) spectrum of **5** in DMSO-d_6	7
11	**Figure S11.** HSQC spectrum of compound **5** recorded in DMSO-d_6	8
12	**Figure S12.** ^{1}H NMR (600 MHz) spectrum of compound **6** recorded in CD_3OD	8
13	**Figure S13.** ^{13}C NMR (150 MHz) spectrum of compound **6** recorded in CD_3OD	9
14	**Figure S14.** HSQC spectrum of compound **6** recorded in CD_3OD	9
15	**Figure S15.** ^{1}H NMR (600 MHz) spectrum of compound **7** recorded in DMSO-d_6	10
16	**Figure S16.** ^{13}C NMR (150 MHz) spectrum of compound **7** recorded in DMSO-d_6	10
17	**Figure S17.** Calibration curve of pick intensity vs concentration of catechin	11

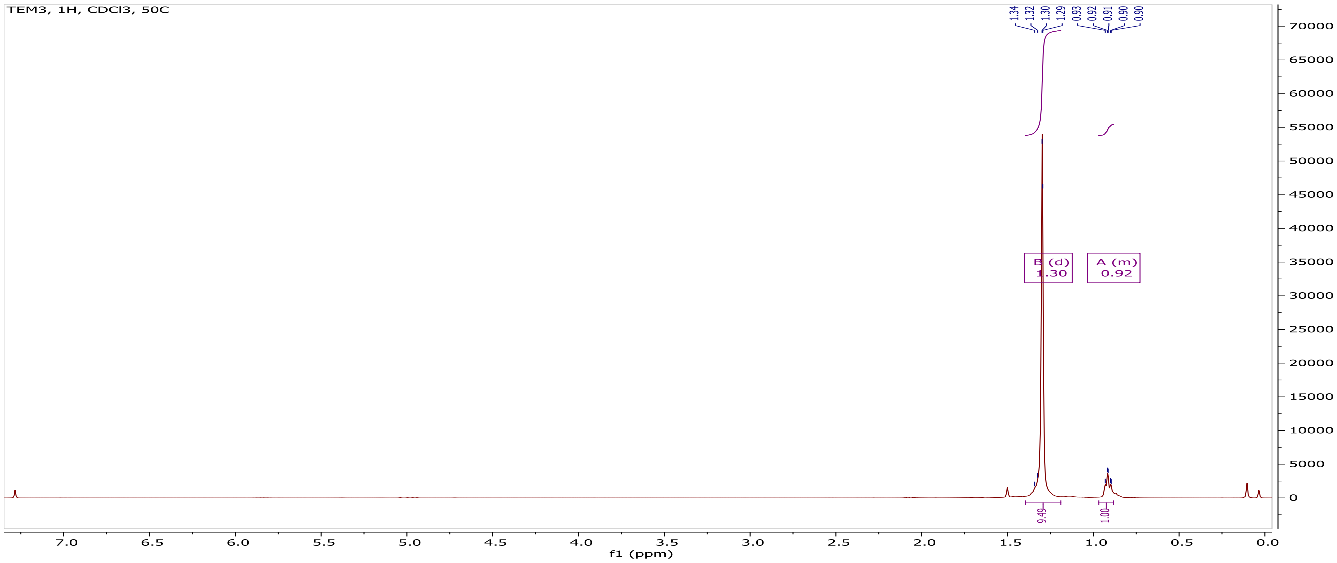

Figure S1. ^{1}H NMR (400 MHz) spectrum of compound **1** recorded in $CDCl_3$

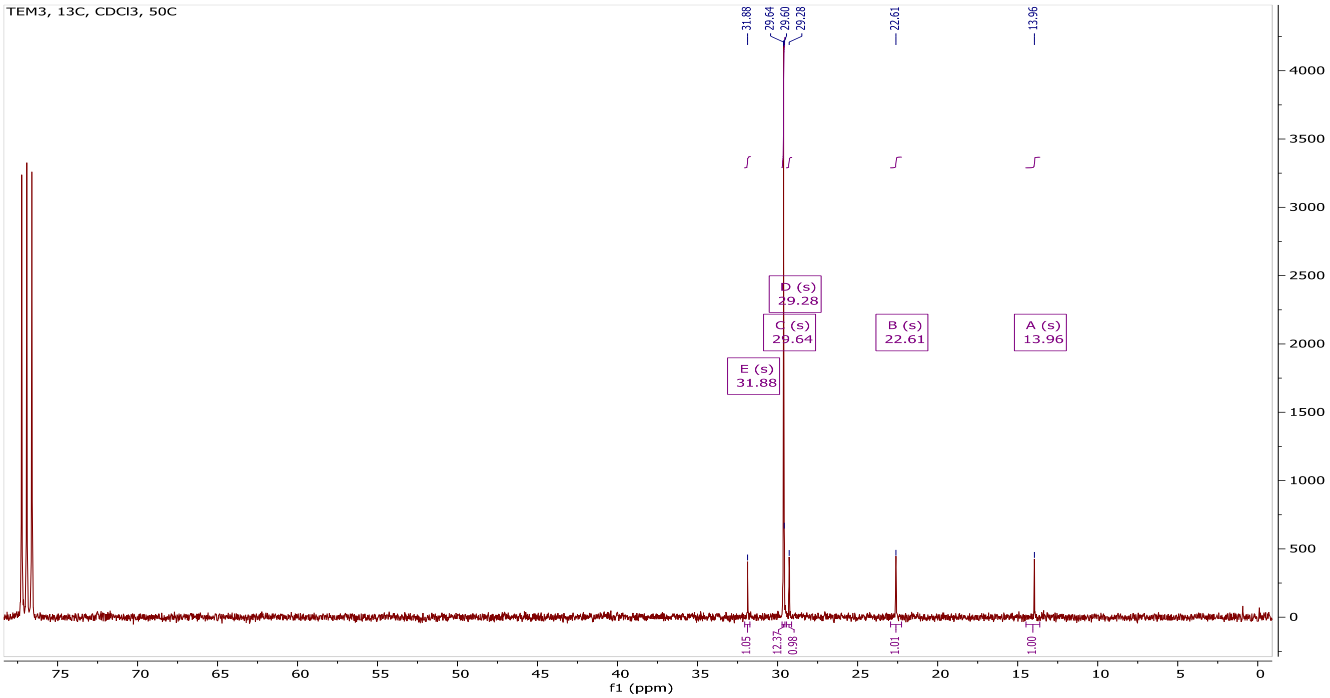

Figure S2. ^{13}C NMR (100 MHz) spectrum of compound **1** recorded in $CDCl_3$

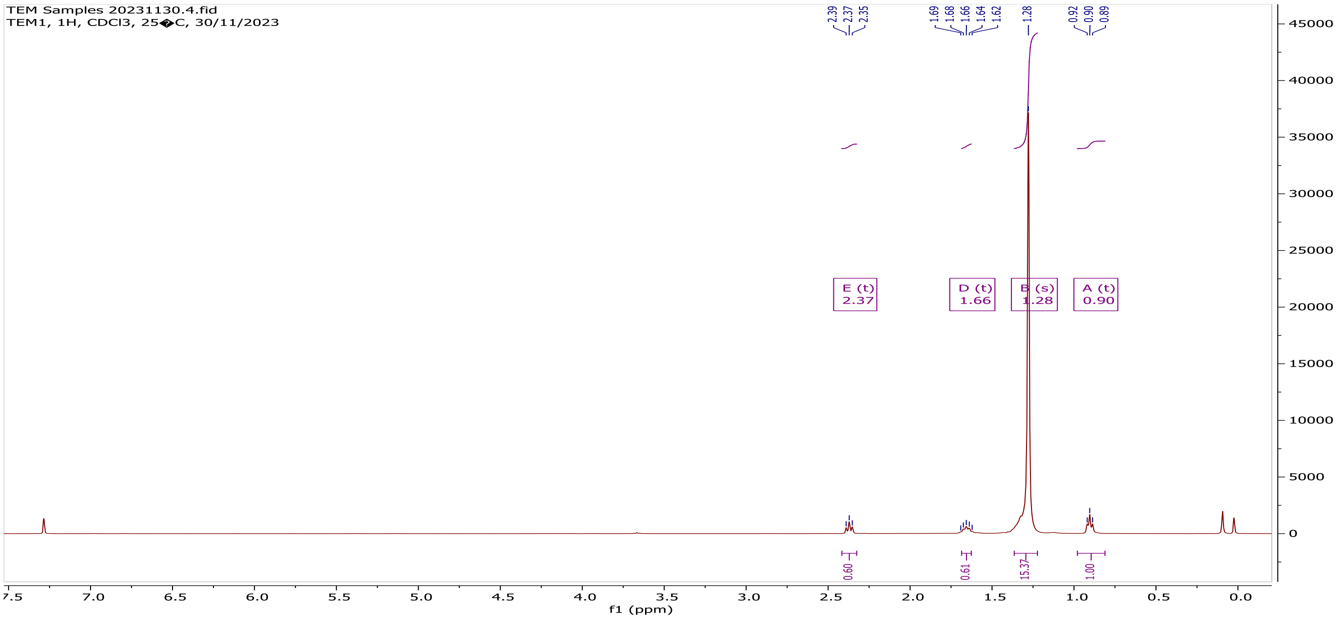

Figure S3. ^{1}H NMR (400 MHz) spectrum of compound **2** recorded in $CDCl_3$

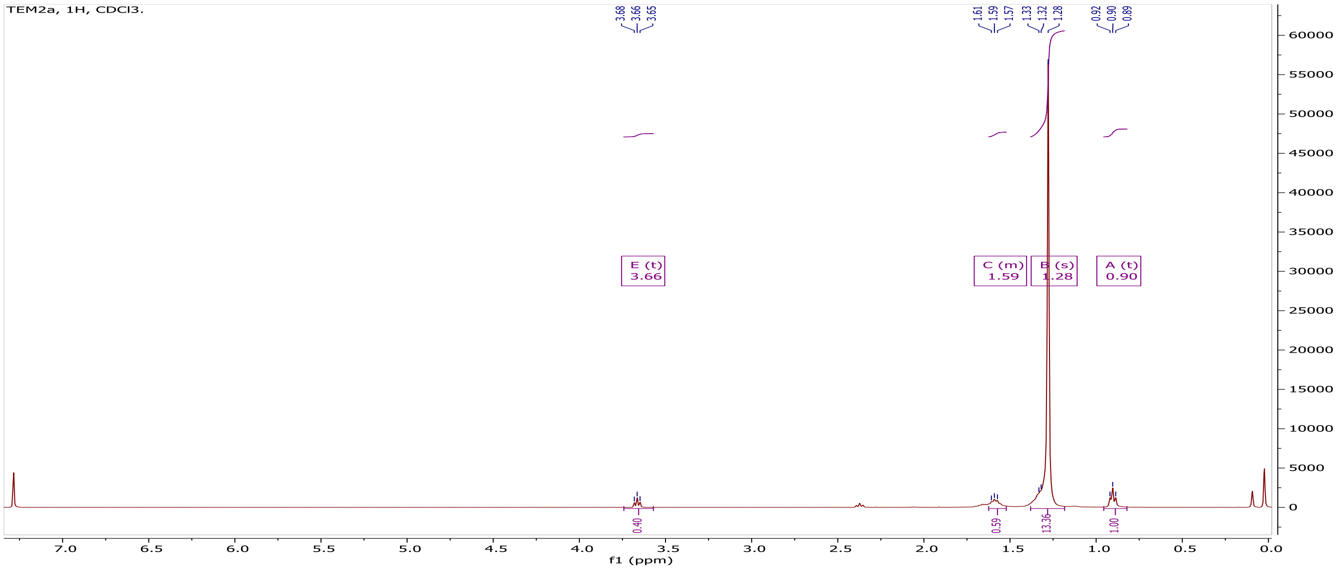

Figure S4. ^{1}H NMR (400 MHz) spectrum of compound **3** recorded in $CDCl_3$

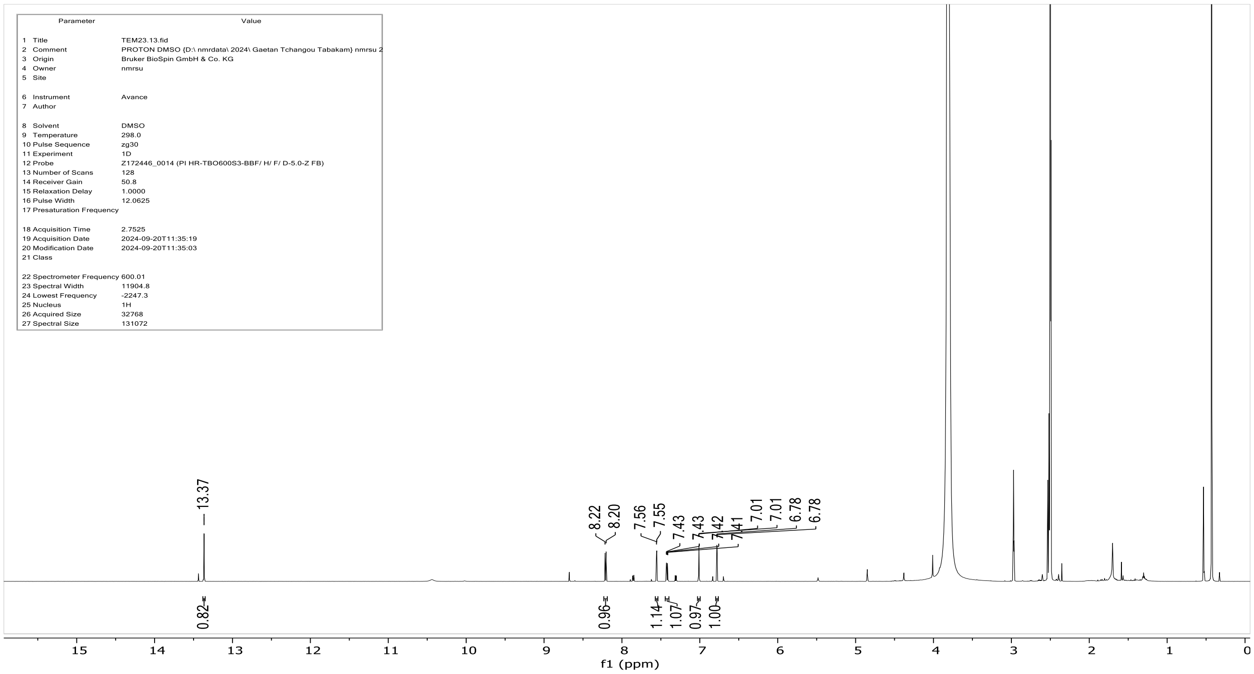

Figure S5. ^{1}H NMR (600 MHz) spectrum of compound **4** recorded in DMSO-d_6

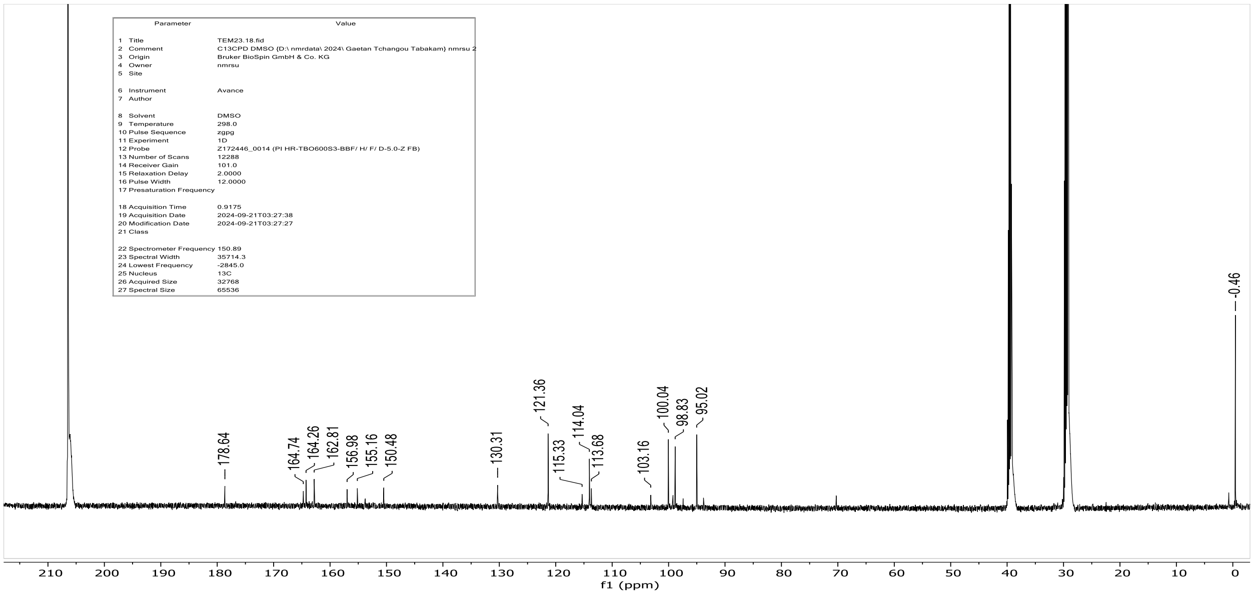

Figure S6. ^{13}C NMR (150 MHz) spectrum of ocmpound **4** recorded in DMSO-d_6

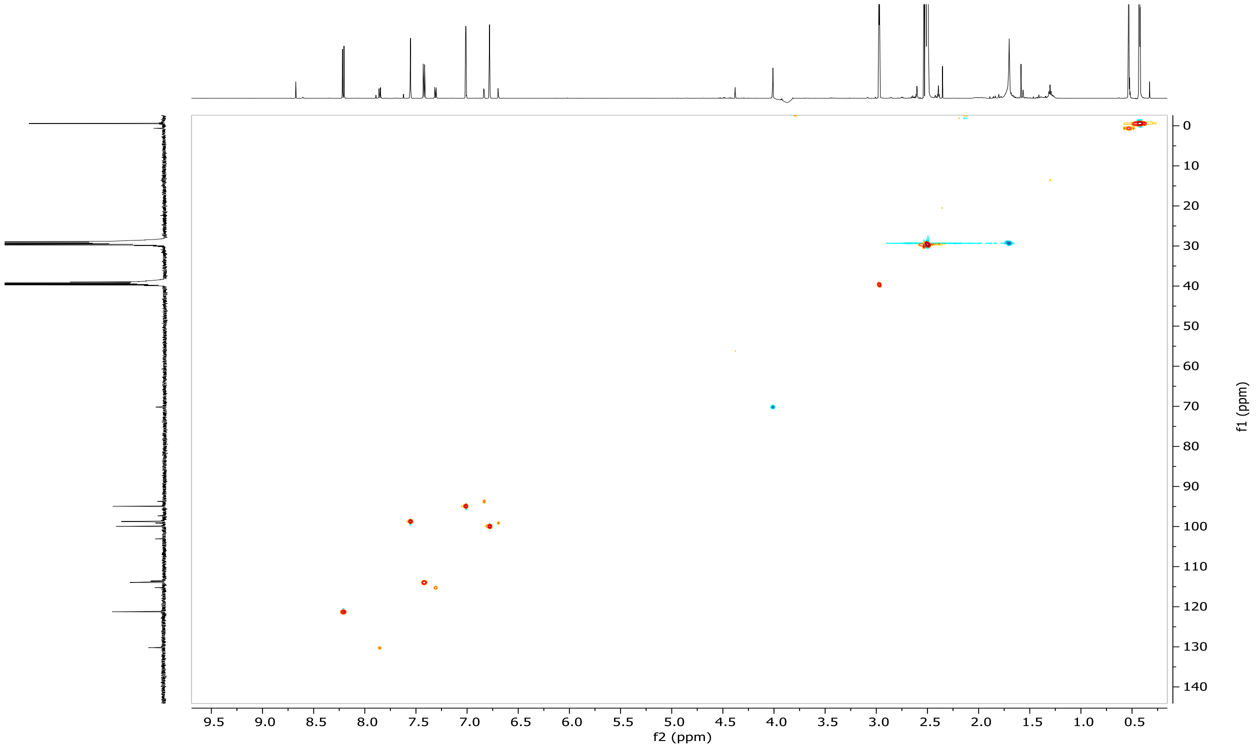

Figure S7. HSQC spectrum of compound **4** recorded in DMSO-d_6

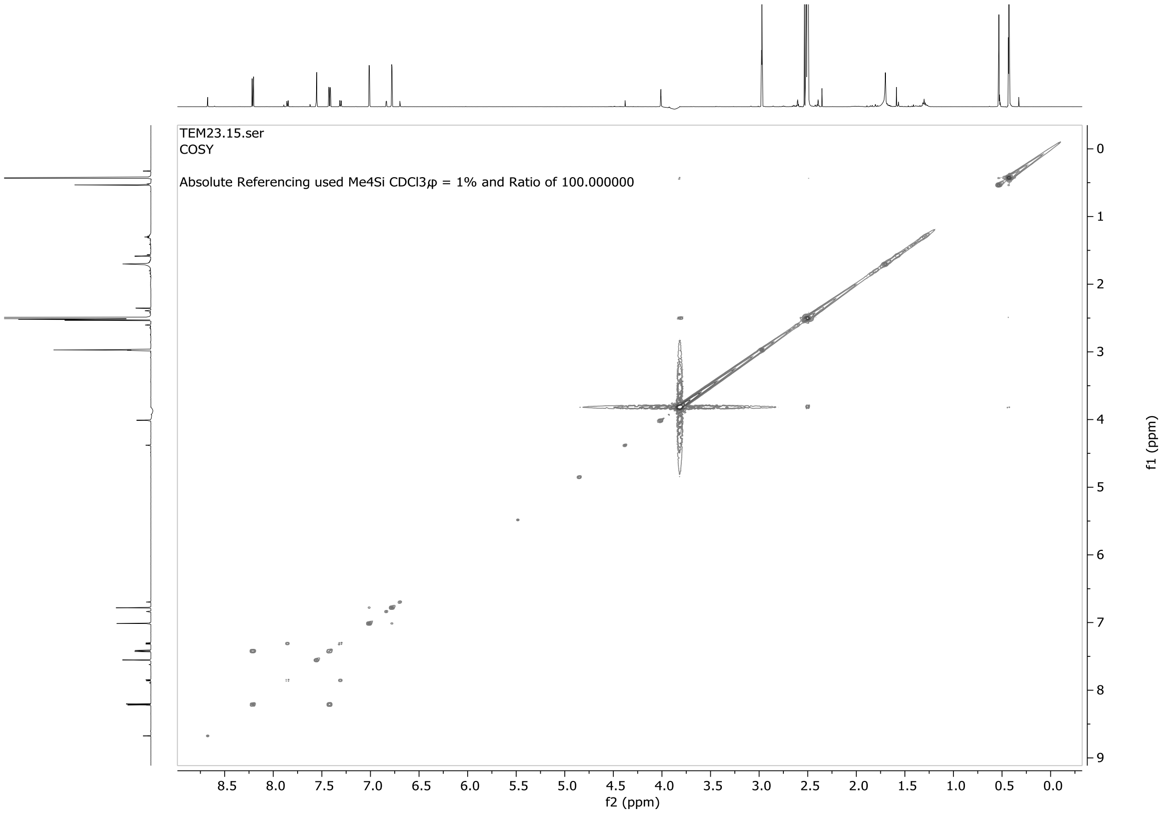

Figure S8. COSY spectrum of compound **4** recorded in DMSO-d_6

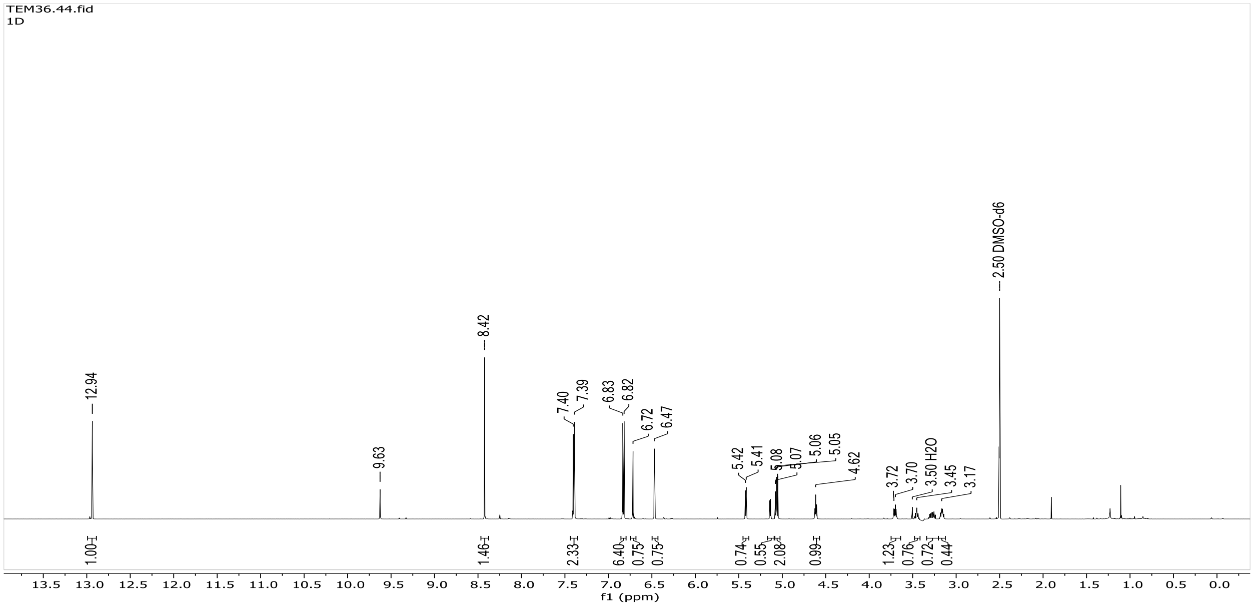

Figure S9. ^{1}H NMR (600 MHz) spectrum of compund **5** recorded in DMSO-d_6

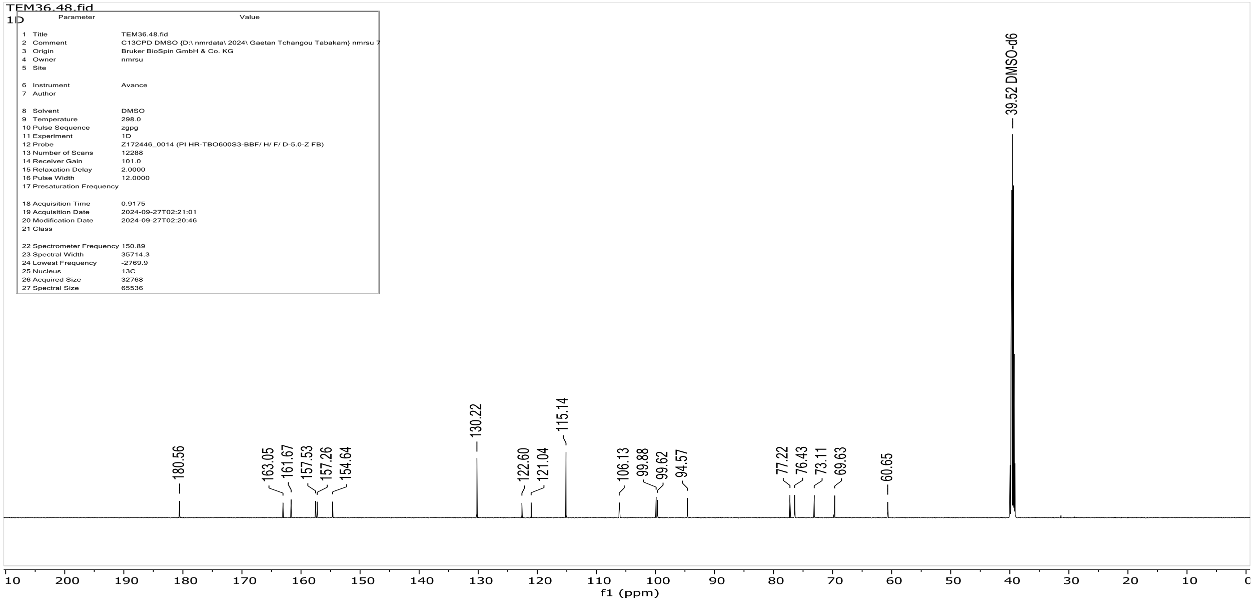

Figure S10. ^{13}C NMR (150 MHz) spectrum of **5** in DMSO-d_6

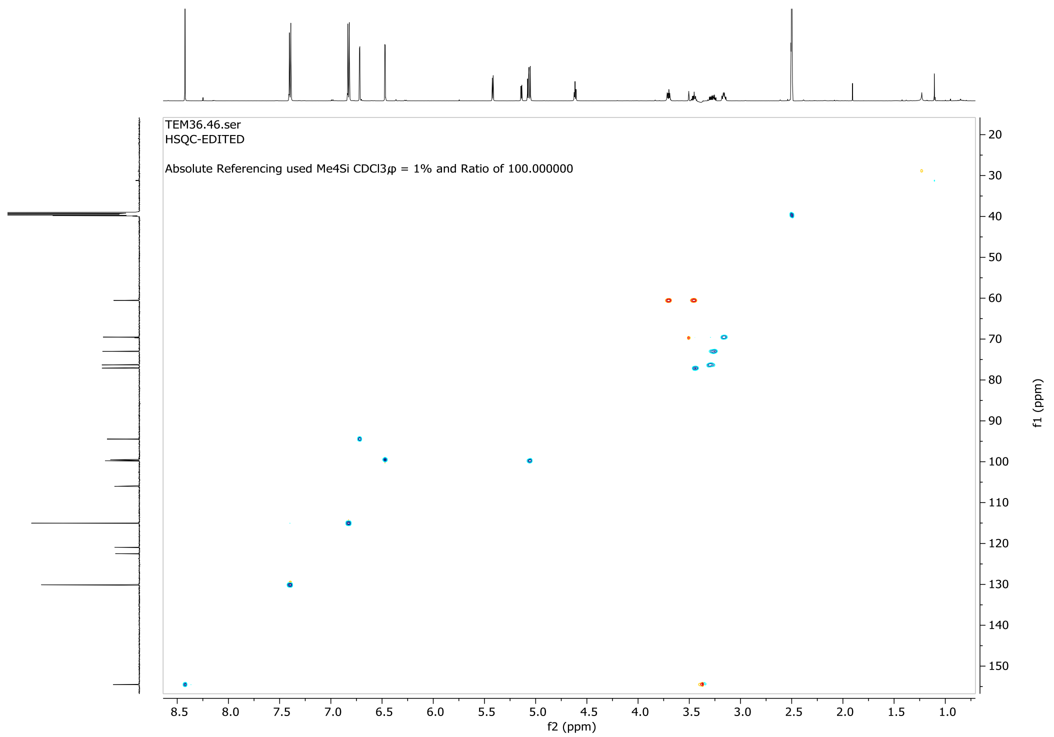

Figure S11. HSQC spectrum of compound **5** recorded in DMSO-d_6

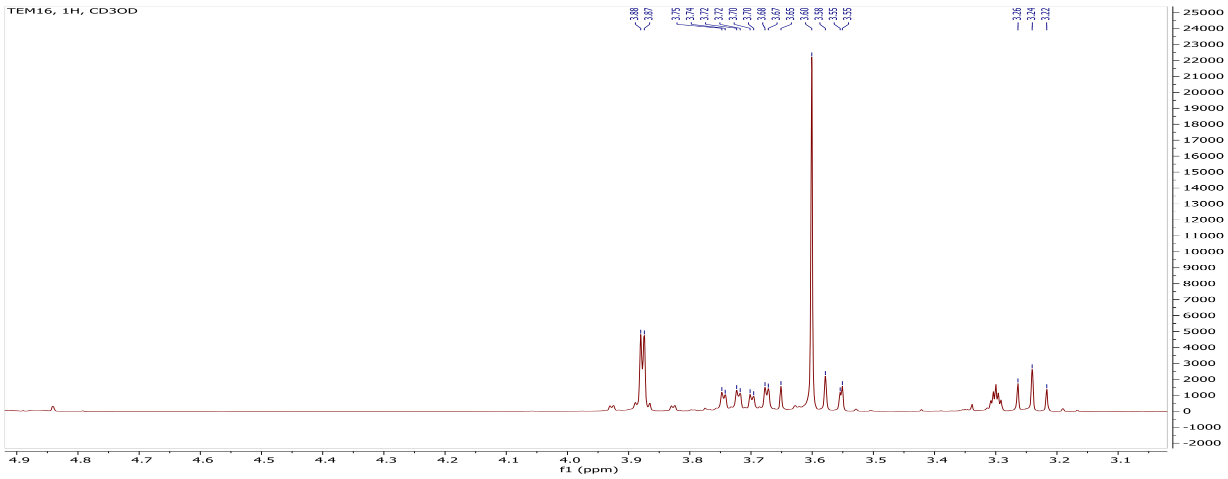

Figure S12. ^{1}H NMR (600 MHz) spectrum of compound **6** recorded in CD_3OD

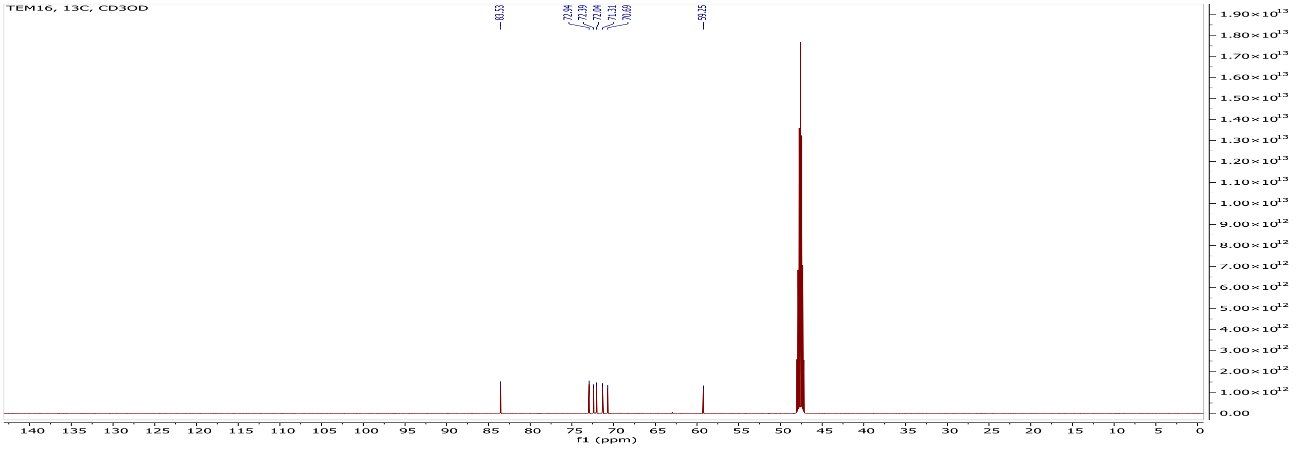

Figure S13. ^{13}C NMR (150 MHz) spectrum of compound **6** recorded in CD_3OD

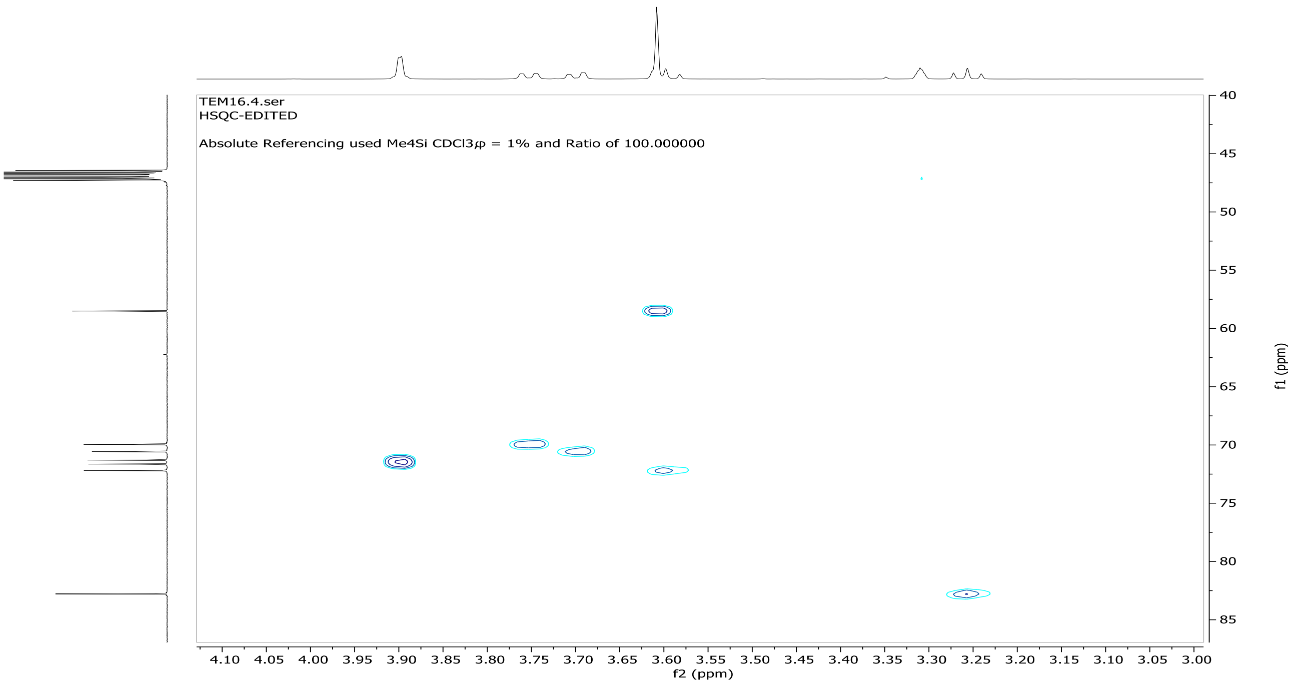

Figure S14. HSQC spectrum of compound **6** recorded in CD_3OD

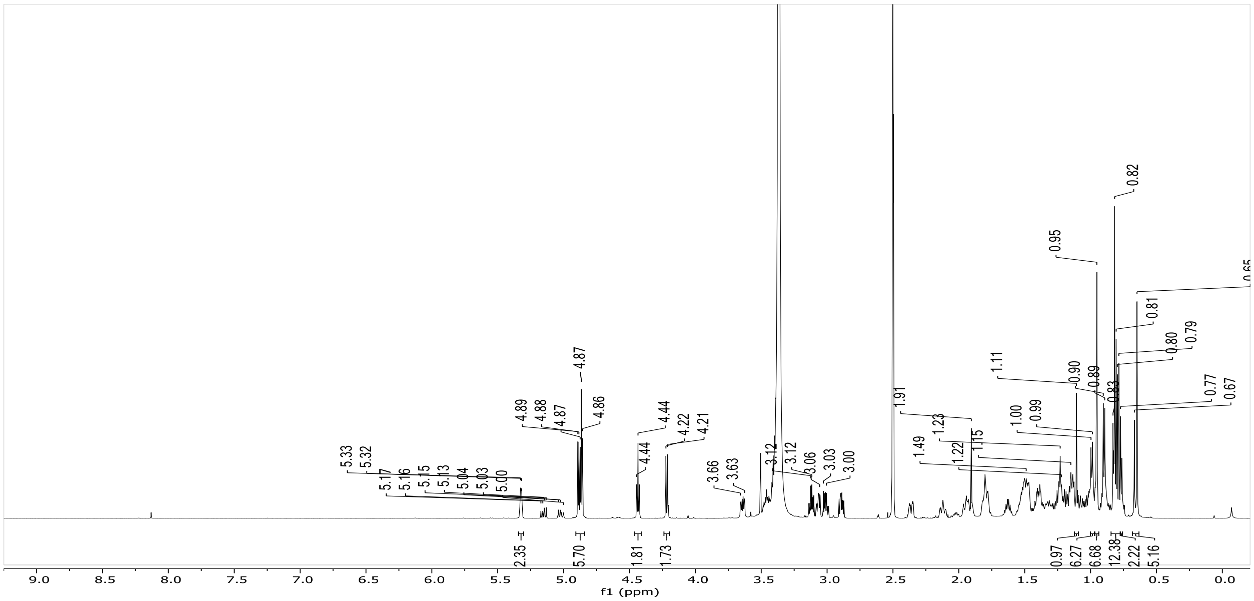

Figure S15. ^{1}H NMR (600 MHz) spectrum of compound **7** recorded in DMSO-d_6

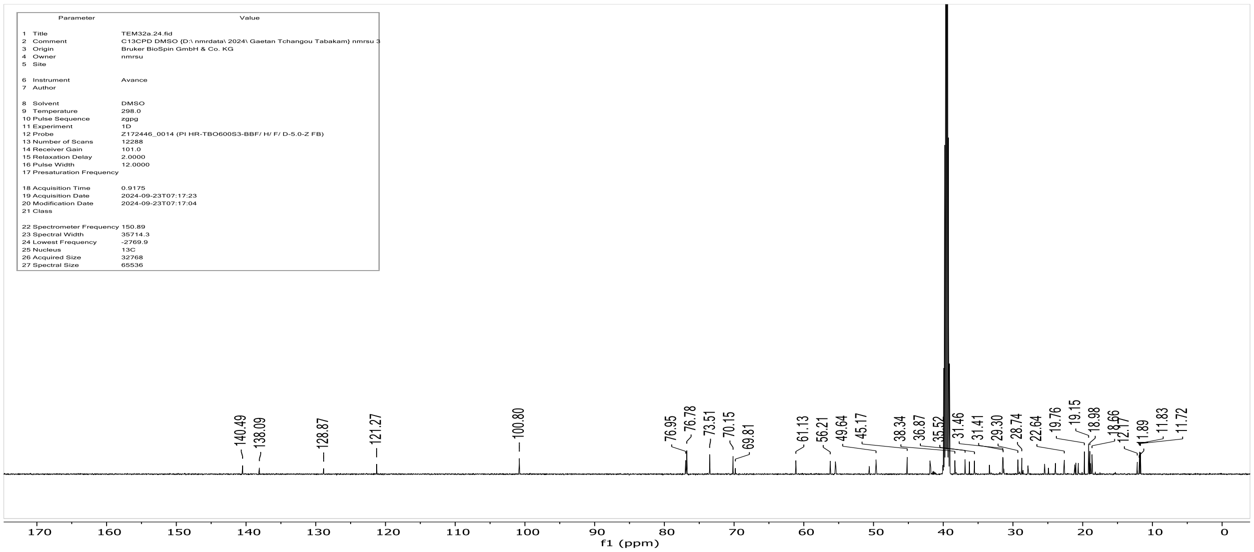

Figure S16. ^{13}C NMR (150 MHz) spectrum of compound **7** recorded in DMSO-d_6

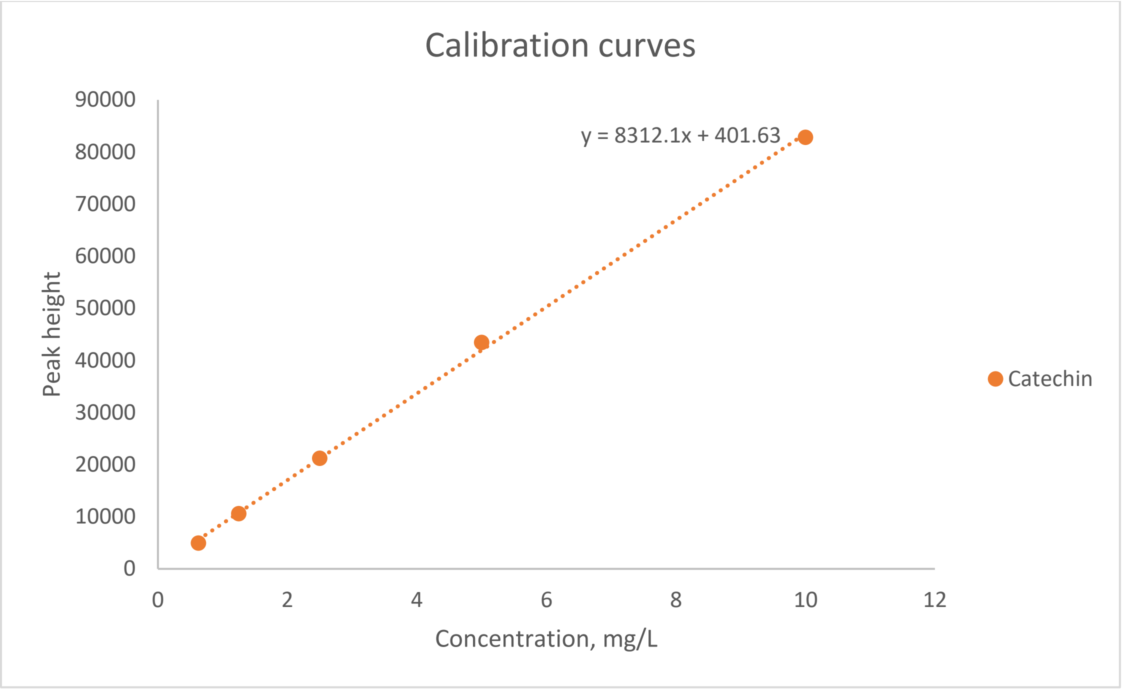

Figure S17. Calibration curve of pick intensity vs concentration of catechin
